# SRF modulates seizure occurrence, activity induced gene transcription and hippocampal circuit reorganization in the mouse pilocarpine epilepsy model

**DOI:** 10.1186/s13041-017-0310-2

**Published:** 2017-07-17

**Authors:** Pascal Lösing, Cristina Elena Niturad, Merle Harrer, Christopher Meyer zu Reckendorf, Theresa Schatz, Daniela Sinske, Holger Lerche, Snezana Maljevic, Bernd Knöll

**Affiliations:** 1 Institute of Physiological Chemistry, Ulm University, Albert-Einstein-Allee 11, 89081 Ulm, Germany; 2 Department of Neurology and Epileptology, Hertie-Institute of Clinical Brain Research, University of Tübingen, Hoppe-Seyler-Straße 3, 72076 Tübingen, Germany; 3 Present address: The Florey Institute of Neuroscience and Mental Health, University of Melbourne, Parkville VIC, Melbourne, 3052 Australia

**Keywords:** SRF, Epilepsy, Pilocarpine, Immediate early gene, Mossy fiber sprouting, Fos

## Abstract

A hallmark of temporal lobe epilepsy (TLE) is hippocampal neuronal demise and aberrant mossy fiber sprouting. In addition, unrestrained neuronal activity in TLE patients induces gene expression including immediate early genes (IEGs) such as *Fos* and *Egr1*.

We employed the mouse pilocarpine model to analyze the transcription factor (TF) serum response factor (SRF) in epileptogenesis, seizure induced histopathology and IEG induction. SRF is a neuronal activity regulated TF stimulating IEG expression as well as nerve fiber growth and guidance. Adult conditional SRF deficient mice (*Srf* ^*CaMKCreERT2*^) were more refractory to initial *status epilepticus* (SE) acquisition. Further, SRF deficient mice developed more spontaneous recurrent seizures (SRS). Genome-wide transcriptomic analysis uncovered a requirement of SRF for SE and SRS induced IEG induction (e.g. *Fos, Egr1, Arc*, *Npas4*, *Btg2*, *Atf3)*. SRF was required for epilepsy associated neurodegeneration, mossy fiber sprouting and inflammation. We uncovered MAP kinase signaling as SRF target during epilepsy. Upon SRF ablation, seizure evoked induction of dual specific phosphatases (*Dusp5* and *Dusp6*) was reduced. Lower expression of these negative ERK kinase regulators correlated with altered P-ERK levels in epileptic *Srf* mutant animals.

Overall, this study uncovered an SRF contribution to several processes of epileptogenesis in the pilocarpine model.

**Electronic supplementary material:**

The online version of this article (doi:10.1186/s13041-017-0310-2) contains supplementary material, which is available to authorized users.

## Introduction

Epilepsy is characterized by repeated seizures as a result of uncontrolled neuronal network synchronization occurring in 1% of the population. Temporal lobe epilepsy (TLE) is the most common form of partial epilepsy which accounts for about 60% of partial epilepsy cases [1, 2]. TLE affected neuronal networks show several molecular and anatomical alterations [3–6]. In resected brain areas of TLE patients, excessive neuronal activity stimulates rapid neuronal gene induction including IEGs [7, 8]. Further, hippocampal sclerosis is a neuropathological hallmark in many patients. This includes granule cell dispersion, loss of pyramidal neurons and immune responses by for instance microglia [9, 10]. Additionally, exuberant mossy fiber sprouting of granule cell neurons is suggested to modulate excitatory or inhibitory properties of seizure-generated neuronal circuits [4].

Several human TLE characteristics are reproduced in rodent models, employing e.g. kainic acid or pilocarpine injection [11, 12]. These epilepsy models replicate IEG induction observed in TLE patients [13–18]. In addition, hippocampal neuronal loss and mossy fiber sprouting are observed [12]. We employed injection of pilocarpine, a cholinergic muscarinic M1 receptor agonist. In this model, mice experience three epilepsy phases, similar to patients. In the acute phase, 20 min - 2 h after injection, mice develop a *status epilepticus* (SE), which lasts for hours unless stopped with anticonvulsants such as diazepam. After experiencing an SE, a latent seizure-free period follows, after which mice enter the chronic phase with frequent spontaneous recurrent seizures [12].

Since exuberant neuronal activity is a characteristic of TLE, we analyzed the neuronal activity regulated transcription factor serum response factor [19–21]. In a previous study, enhanced SRF occupancy at target genes was reported in the rat pilocarpine model [22]. However, so far, an impact of SRF on gene expression, seizure occurrence and histo-pathological seizure hallmarks was not investigated in the pilocarpine model. SRF mediates IEG responses elicited by physiological [23–27] and pathological stimuli [22, 28]. In the kainic acid model, SRF mediates an SE triggered IEG response [22]. In addition, SRF occupancy at IEG promoters [22, 29] and SRF phosphorylation [30], indicative of SRF activation, are enhanced in mouse epilepsy models. Here, SRF might team up with its Elk-1 cofactor whose ablation reduces IEG induction in epileptic mice [31]. In adult *Srf* mouse mutants, SRF ablation enhanced SRS frequency [22], but so far no impact of SRF on the initial SE was reported in any model. Herein, we show for the first time the role of SRF in pilocarpine-evoked SE occurrence. Similarly, SRF was so far not associated with epileptiform hippocampal sclerosis, despite reported functions in related processes such as neurodegeneration [32, 33], axon growth, pathfinding and regeneration [34–39] as well as inflammation [40]. Employing adult SRF ablation in glutamatergic forebrain neurons (*Srf* ^*CaMKCreERT2*^), we show here decreased epilepsy associated neurodegeneration, mossy fiber sprouting and inflammation.

SRF is activated through MAP kinase signaling, a crucial signaling pathway transducing seizure activity [6]. ERK and ERK inhibiting phosphatases, so-called DUSPs (dual specific phosphates), are activated in rodent seizure models [41–44] and epilepsy patients [7]. Moreover, ERK inactivation [45, 46] or activation [47] results in decreased and enhanced seizure activity, respectively. Our study suggests a feedback loop of SRF with MAP kinase signaling underlying SRF’s role in epileptic mice.

In summary, we show a role for SRF in epilepsy associated gene transcription, neurodegeneration, mossy fiber sprouting and inflammation.

## Methods

### Mice and tamoxifen injection

*Srf* ^*CaMKCreERT2*^ mice were described before [40]. All mice were on a C57/Bl6 background. In brief, mice carrying a floxed *Srf* allele [48] were interbred with CaMKCreER^T2^ mice obtained from G. Schütz [49]. In general, we used male and female littermates derived from breeding pairs with both parents bearing the genotype *Srf* ^+/−^ and CreER^T2^-positive for all experiments. We did not observe differences in seizure responses between males and females. As control mice (control), we used for all experiments animals being heterozygous (het) for *Srf* and positive for the Cre allele (i.e. *Srf* ^*+/−;CaMKCreERT2*^). For the Cre allele, we cannot distinguish between mice harboring a single or two Cre alleles. After Cre mediated recombination, SRF-deficient animals were designated *Srf* ^*−/−;CaMKCreERT2*^. After seven to 8 weeks after birth, we injected all animals used for experiments with tamoxifen (2 mg dissolved in 10% ethanol in peanut oil; Sigma) once a day for five consecutive days.

The local governmental authority for animal experimentation (Regierungspräsidium Tübingen, Germany) approved all of the mouse experiments in this study.

### Seizure induction

Seizures were induced by administration of pilocarpine, a muscarinic cholinergic agonist [50, 51]. In brief, 20 min before pilocarpine administration, animals were injected with a low dose of the cholinergic antagonist methyl scopolamine nitrate (1 mg/kg, s.c.; Sigma, Germany) to reduce peripheral cholinergic effects. Animals received an intra-peritoneal injection of pilocarpine hydrochloride (335 or 350 mg/kg; Sigma, Germany) to induce an SE. Littermate control mice were injected with Ringer solution. Diazepam (4 mg/kg, s.c.; Ratiopharm, Germany) was administered to the animals 40 min after the onset of SE to terminate seizures. All drugs were dissolved in Ringer solution. After pilocarpine injection, all animals were observed for a time period of 4 h to assess severity and duration of behavioral seizures. Pilocarpine-induced seizures were classified based on the Racine scheme as described before [40], i.e. stage 1: immobility, rigid posture; stage 2: repetitive movements, head bobbing; stage 3: motor seizure with forelimb clonus; stage 4: severe seizures with rearing without falling; stage 5: severe seizures with rearing and falling or jumping; SE was defined by continuous seizure activity for at least 10 min without full recovery between seizures. In this study, mice were either killed after 40 min of SE (40 min of continuous seizure activity without full recovery between seizures) or injected with diazepam. After SE, all mice were fed with 5% glucose solution (Fresenius, Germany) and soaked rodent food.

### EEG recordings

For long term seizure and EEG monitoring, a permanent telemetric EEG−/video-monitoring system (Data Science International, USA) was used. Nine days before SE induction, mice were implanted with EEG electrodes using stainless steel screws positioned at posterior −1.5, lateral −1.5 in millimeter relative to bregma under deep anesthesia (6 mg/kg Xylazin, Albrecht, Germany and 90–120 mg/kg Ketamin, WDT Germany). The electrodes were fixed with cement. The EEG transmitter was subcutaneously placed on the right abdominal side and connected to the electrodes subcutaneously. All implanted mice received analgesic treatment for 2 days (5 mg/kg Ketoprofen, Gabrilen, Mibe, Germany, s.c. twice a day) after implantation as well as antibiotic treatment during the monitoring phase (5 mg/kg p. o. Enrofloxacin, Baytril®, Bayer, Germany). Two days after implantation, video/EEG monitoring was started to allow for baseline recording before seizure induction. Nine days after EEG electrode implanting, pilocarpine injection to induce SE was performed as described above. The sampling rate of EEG recordings was 500 Hz and they were analyzed using fast Fourier power spectral analysis (FFT), provided by Neuroscore 3.0 software (Data Science International, USA). Band power was analyzed in five frequency bands: delta (0.5–3.99 Hz), theta (4–7.99 Hz), alpha (8–12 Hz), beta (16–24 Hz), and gamma (30–40 Hz). The relative band power increase was calculated by normalizing the area under the curve of each power band from the period of pilocarpine injection until diazepam injection to the corresponding area under the band power curve for baseline EEG signal of identical duration obtained 24 h before SE. From concurrent video recordings, all spontaneous seizures were classified as described previously [51]. In brief, a stage 4 SRS consisted of severe seizures with rearing but without falling whereas a stage 5 SRS was scored when severe seizures with rearing and falling or loss of righting ability occurred. EEG spikes were detected using an absolute threshold protocol with a set threshold value of 100 μV to a maximum of 5000 μV. The minimum spike duration was set to 5 ms and maximum spike duration to 70 ms. A spike train was defined by a minimum spike interval of 0.005 s and a maximum spike interval of 1 s. Minimum train duration was set to 30 s.

Of note, visual inspection of seizure occurrence (see above) was performed at Ulm University, whereas EEG recordings were performed at Tübingen University.

### Transcriptomics and quantitative real-time PCR (qPCR)

For RNA isolation from mouse hippocampal tissue, we employed the ISOLATE II RNA/DNA/Protein kit (Bioline) or the mini RNeasy kit (Qiagen) according to manufacturer’s instructions. Reverse transcription was performed with 1 μg RNA using reverse transcriptase (Promega) and random hexamers. We performed qPCR on the Light Cycler 480II (Roche) with the Power PCR SYBR green PCR master mix (Takara). Typically, 2 μl of cDNA were used in a 10 μl reaction volume/well of a 96-well plate. Doublets were performed for each sample. The LC480 II Software detects this threshold cycle value (Ct value) for each sample. The higher the Ct value, the lower the original cDNA amount for a certain gene in the sample. In order to neutralize potential variations in total mRNA amounts used for the cDNA synthesis, the Ct values of the house-keeping gene *Gapdh* (glycerinaldehyd-3-phosphat-dehydrogenase). *Gapdh* is a well-suited house-keeping gene for normalization in our experiments since the *Gapdh* mRNA abundance was not altered by seizure induction (data not shown and see Additional file 1: Table S1) in line with a previous report [52]. Normalization was performed according to the following equation: relative mRNA expression = 2^-ΔCt (ΔCt = Cttarget gene - CtGapdh)^. Primer sequences are provided upon request.

For transcriptomics, a total of 18 samples (het ctr.: *N* = 3; het + SE: *N* = 3; mut ctr: *N* = 3; mut -SE: *N* = 3; mut + SE: *N* = 2; het -SRS: *N* = 1; het + SRS: *N* = 1; mut –SRS: *N* = 1; mut + SRS: *N* = 1) derived from 18 mice were subjected to microarray analysis. 100 ng total RNA was used as starting material and 5.5 μg ssDNA per hybridization (GeneChip Fluidics Station 450; Affymetrix, Santa Clara, CA). The total RNAs were amplified and labeled following the Whole Transcript (WT) Sense Target Labeling Assay (http://www.affymetrix.com). Labeled ssDNA was hybridized to Mouse Gene 1.0 ST Affymetrix GeneChip arrays (Affymetrix, Santa Clara, CA). The chips were scanned with an Affymetrix GeneChip Scanner 3000 and subsequent images analyzed using Affymetrix® Expression Console™ Software (Affymetrix). Raw feature data were normalized and intensity expression summary values for each probe set were calculated using robust multiarray average [53]. Microarrays were analyzed with microarray Я US and Limma 2way Anova with *p*-Value and FDR cutoffs at 0.001 and 2 fold change cut-off. All microarray data were deposited at the Gene Expression Omnibus (GEO) repository (GSE100202).

### Chromatin immunoprecipitation (ChIP)

Anti-SRF directed ChIP was performed as described in [54].

### Histology

We fixed mouse brains in 4% formaldehyde (FA) followed by preparation of 5 μm paraffin microtome slices. Immunohistochemistry was performed using Biotin conjugated secondary antibodies (1:500; Vectorlabs, Lörrach, Germany) and peroxidase based detection systems using the ABC complex (Vectorlabs) and DAB as substrate. Alternatively, Alexa488 or 546 (1:500; Life Technologies, Darmstadt, Germany) conjugated secondary antibodies were used. Primary antibodies included anti-SRF (rabbit, 1:2500; Santa Cruz, Heidelberg, Germany), anti-IBA (rabbit, 1:1000; Wako Chemicals, Neuss, Germany), anti-Egr1 (rabbit 1:500; Santa Cruz), Fos (rabbit, 1:500; Santa Cruz), anti-NeuN (1:1000; Millipore, Billerica, USA), anti-ZnT3 (1:2500; Synaptic Systems, Göttingen, Germany), anti-P-ERK (1:1000; Cell Signaling, Cambridge, UK) and anti-P-MEK (1:1000; Cell Signaling).

#### Fluoro-Jade B (FJB) staining

Mouse brains were fixed in 4% FA, embedded in paraffin and cut in 5 μm slides. Sections were stained as previously reported [55]. In brief, slides were treated for 10 min with Xylene followed by 3 min 100% Ethanol, 1 min of 70% Ethanol, 30% Ethanol and H_2_O. Background was reduced by treatment with 0.06% KMnO4 in H_2_O for 15 min and subsequently washed for 1 min in H_2_O. FJB staining was performed for 30 min with 0.001% FJB (Millipore, Billerica, USA), 0.001% acetic acid, 0.0002% DAPI. Stained slides were washed 3 times for 1 min in H_2_O, dried and mounted in Entellan.

### Biochemistry

Protein lysates were prepared with the ISOLATE II RNA/DNA/protein kit (Bioline) according to manufactures instructions. 1× PhosStop (Roche) was added to the protein lysates. Samples were resolved on 8–10% SDS-PAGE, followed by transfer on PVDF membranes (Amersham). After 1 h of blocking, first antibodies were applied overnight at 4 °C: rabbit anti-P-ERK (1:1000, Cell Signaling), rabbit anti-ERK (1:1000; Cell Signalling), rabbit anti-Elk-1 (1:1000; Cell Signalling) and rabbit anti-P-CREB (1:1000; Cell Signalling). Detection of first antibodies involved horseradish-peroxidase conjugated secondary antibodies (1:2000; Santa Cruz) and the ECL Western Blotting Substrate (Pierce or Millipore).

### Cell culture

P3–5 mouse cerebellar neurons were plated on poly-L-lysine (PLL; 100 μg/ml) and laminin (5 μg/ml) coated wells of a 6-well plate. Electroporation with SRF-VP16 expressing constructs was performed with 3 μg DNA and 100 μl electroporation solution (Mirus) as before [34].

### In silico analysis of potential SRF binding sites, Venn analysis and STRING networks

Overrepresentation analysis of transcription factor binding sites (TFBS) was performed using Pscan Ver. 1.3 [56] using all at least four-fold upregulated genes after SE or 1 h after SRS respectively. For Venn diagrams Venny 2.1, an interactive tool for comparing lists with Venn diagrams was applied (http://bioinfogp.cnb.csic.es/tools/venny/index.html). For STRING analysis the following software package was used [57].

### Statistical analysis and quantification

Numbers (*N*) of independent cell cultures or animals were indicated in figure bars or text. For statistical analysis of data GraphPad Prism software (GraphPad Software, Inc.) was used. Unpaired t test or Mann–Whitney test (nonparametric) was used for comparing two groups, whereas two-way analysis of variance (ANOVA) with post hoc Bonferroni’s multiple comparisons test was used for comparison of multiple groups. Statistical significance is provided as *, **, *** indicating *p* ≤ 0.05, 0.01 and 0.001, respectively. Standard deviation (s.d.) is provided if not mentioned otherwise.

## Results

### Characterization of *Srf* mutant animals

**Fig. 1:**
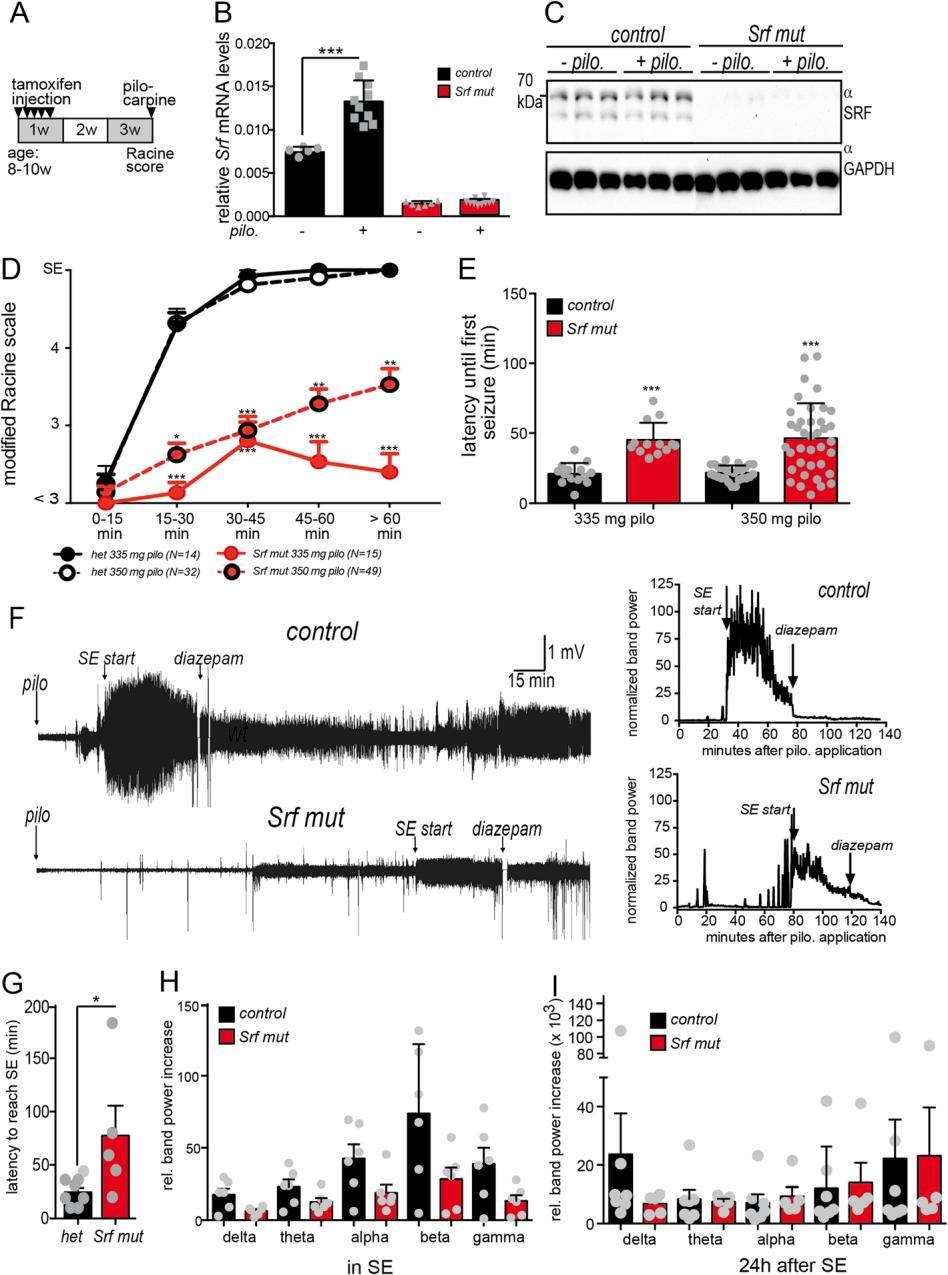
SRF ablation reduces occurrence of SE and SE intensity. **a** Experimental set-up. Mice were injected five times with tamoxifen, followed by 14 days allowing for SRF downregulation. Subsequently, pilocarpine was injected and mice were analyzed. **b**, **c** Hippocampal samples were analyzed for *Srf* mRNA (**b**) or SRF protein (**c**) abundance. In heterozygous mice being 40 min in SE, *Srf* mRNA (**b**) but not SRF protein (**c**) was elevated by pilocarpine injection at this early time-point. *Srf* mRNA (**b**) or protein (**c**) was clearly reduced in *Srf* mutant mice. **d** Mice were injected with 335 or 350 mg/kg pilocarpine and seizure intensity was scored every 15 min by the Racine scale in the first 2 h. All heterozygous control mice entered SE status after approximately 45 min for both pilocarpine concentrations. In contrast, in mutant mice the seizure score was significantly reduced for both pilocarpine concentrations and no animal reached SE after 45 min with a dose of 335 mg/kg pilocarpine. Data are represented as mean ± SEM. **e** The latency until the first seizure was almost doubled in *Srf* mutant compared to heterozygous mice for both pilocarpine concentrations. **f** Representative raw (*left*) and normalized (*right*) EEG recordings of one heterozygous and one *Srf* mutant animal depicting start of pilocarpine injection, SE start and diazepam injection after 40 min of SE. **g** In SRF deficient mice, it takes longer until SE is achieved after pilocarpine injection. **h** To quantify SE severity, the area under the different *band power curves* was determined from SE initiation until diazepam injection. The values were normalized to the area under the band power curve obtained from baseline EEG recordings of identical duration obtained 24 h before SE. In control animals all frequency bands during SE were elevated compared to *Srf* mutant mice, however not in a statistically significant manner. **i** 24 h after diazepam injection heterozygous and SRF deficient mice had comparable EEG activities. Data are represented as mean ± SD. Number of animals are indicated or individual animals are labeled with *grey circles*

In this study we employed forebrain specific deletion of SRF in glutamatergic neurons via a tamoxifen inducible Cre recombinase driven by *Camk2a* promoter [40]. At the age of seven to 8 weeks, mice were injected five times with tamoxifen and analyzed 2 weeks thereafter (Fig. 1a). After tamoxifen induced Cre activity, mice harboring two floxed *Srf* alleles were designated as *Srf* mutants (*Srf* ^*loxp/loxp; CaMKCreERT2*^ or *Srf mut*). As control mice, heterozygous (het) littermates with one *Srf* allele and harboring at least one *Cre* allele were used (*Srf* ^*loxp/+; CaMKCreERT2*^). We compared *Srf* ^*+/+; CaMKCreERT2*^ and *Srf* ^*loxp/+; CaMKCreERT2*^ mice to see whether loss of one *Srf* allele in heterozygous mice already has an impact on seizure occurrence and gene expression. In heterozygous mice, the SRF protein levels were slightly reduced compared to *Srf* ^*+/+; CaMKCreERT2*^ mice [58]. Upon pilocarpine injection, no differences between both genotypes were observed in the Racine scale scoring and all mice acquired SE status at almost identical times (*N* = 5; data not shown). Similarly, in a previous report, we did not observe differences between wildtype and heterozygous mice. Here, hyperactivity and anxiety-related phenotype as well as impaired gene expression was only observed in *Srf* mutant but not in heterozygous mice [58]. This suggests that heterozygous mice behave comparably to mice harboring two wildtype (wt) alleles and can be used as controls.

Tamoxifen mediated Cre recombinase activity resulted in robust *Srf* mRNA (Fig. 1b) and SRF protein (Fig. 1c) ablation in *Srf* mutant mice compared to heterozygous littermates in the hippocampus (Fig. 1b, c). In addition, SRF is depleted from the cortex and striatum [40]. In control mice experiencing an SE for 40 min, *Srf* mRNA levels were elevated (Fig. 1b). In contrast, no obvious effect on SRF protein abundance was observed at this early time-point (Fig. 1c). The latter might be explained by the short SE duration of 40 min that did not allow for maximal SRF protein upregulation yet. Indeed, at later time-points, i.e. 24 h after an SE enhanced SRF promoter occupancy was reported [29].

### SRF ablation reduces SE occurrence upon pilocarpine injection

In a previous report, consequences of SRF ablation upon intra-hippocampal kainic acid injection were analyzed. Here, *Srf* mutants showed elevated SRS frequency [22]. However, acquisition of an initial kainic acid induced SE phase is comparable in *Srf* mutant and heterozygous mice, suggesting that SRF is not required for SE occurrence [22]. In accordance, intra-peritoneal kainic acid injection performed in this study also did not affect SE occurrence (Additional file 2: Figure S1).

Since data reported so far in kainic acid model did not show a requirement of SRF for SE development, we turned to the pilocarpine model and investigated whether SRF ablation interfered with SE occurrence in this epilepsy model (Fig. 1). For this, mice were intra-peritoneally injected with an established pilocarpine concentration of 335 mg/kg bodyweight (Fig. 1d, e). Subsequently, seizures were scored according to the modified Racine scale for four hours. All control mice reached SE within 45 min after pilocarpine injection (*N* = 14 out of 14 mice; Fig. 1d). After 40 min in SE, mice were injected with diazepam to reduce seizure intensity. In contrast to heterozygous control mice, none of the 15 SRF deficient mice reached the SE stage after 45 min (Fig. 1d). Even 2 h after pilocarpine injection, none of the mutant mice reached SE (data not shown). Next we increased the pilocarpine doses to 350 mg/kg which induced SE in all control mice (*N* = 32 out of 32 mice; Fig. 1d). In contrast, only one third of SRF deficient mice reached SE after 60 min (*N* = 15 out of 49 mice; Fig. 1d). Of note, although *Srf* mutant mice had decreased SE occurrence, mice responded to pilocarpine with shaking and head nodding resulting in lower Racine scores of around two to three (Fig. 1d). We next measured the latency to the first seizure response (Fig. 1e). *Srf* mutant mice showed an approximately two-fold increased latency until a first seizure response of at least stage 4 was observed (Fig. 1e).

In addition to scoring seizures visually by the Racine scale (Fig. 1d, e), a permanent telemetric EEG/video monitoring procedure was applied (Fig. 1f-i). The EEG electrodes were implanted 9 days before SE induction to allow for a recovery from surgery and a prolonged period of baseline EEG recordings. No obvious differences in baseline activity during EEG recordings without pilocarpine injection were observed between control and *Srf* mutant mice (data not shown). Also, in EEGs we did not observe spontaneous seizure occurrence in *Srf* mutant mice without pilocarpine administration (data not shown).

In EEG experiments, all heterozygous control animals (*N* = 8) experienced an SE period starting approximately 30–45 min after pilocarpine injection (Fig. 1f). In *Srf* mutant animals, the combination of electrode implantation and pilocarpine injection increased lethality. Thus, in EEG experiments only six out of 13 *Srf* mutant animals reached an SE stage. The other seven animals died (*N* = 4) or did not reach SE status (*N* = 3). This corroborated results obtained with Racine scale scoring (see above; Fig. 1d, e). In those *Srf* mutant mice reaching SE, the time until reaching SE was increased compared to control mice (Fig. 1f; quantified in g). These results suggest decreased seizure occurrence upon SRF ablation. Next, we quantified seizure intensity by calculating the area under the band power curves for each frequency band. During the SE period, all frequency bands showed strongly elevated levels relative to the baseline values obtained before pilocarpine administration. After 40 min in SE, this period was terminated with diazepam, resulting in substantially decreased activity (Fig. 1f). EEG activity in all band frequencies was elevated in heterozygous compared to *Srf* mutant animals, however no statistically significant difference in any single band was observed between experimental groups (Fig. 1h). This lack of statistical significance might indicate an overall comparable SE intensity between control and SRF deleted animals. However, despite this lack of statistical significance, the relative band power increase was reduced by approximately 50% in all bands in SRF deficient mice compared to control mice (Fig. 1h). Thus, we cannot completely rule out a decreased EEG intensity during the SE period upon SRF ablation and this has to be taken into account for data interpretation.

One day after reducing SE intensity by diapezam injection, EEG activity was indistinguishable for most bands between heterozygous and *Srf* mutant animals (Fig. 1i).

Overall, this is a first result reporting an interference of SRF ablation with SE acquisition.

### Pilocarpine mediated gene expression is impaired upon SRF ablation

**Fig. 2:**
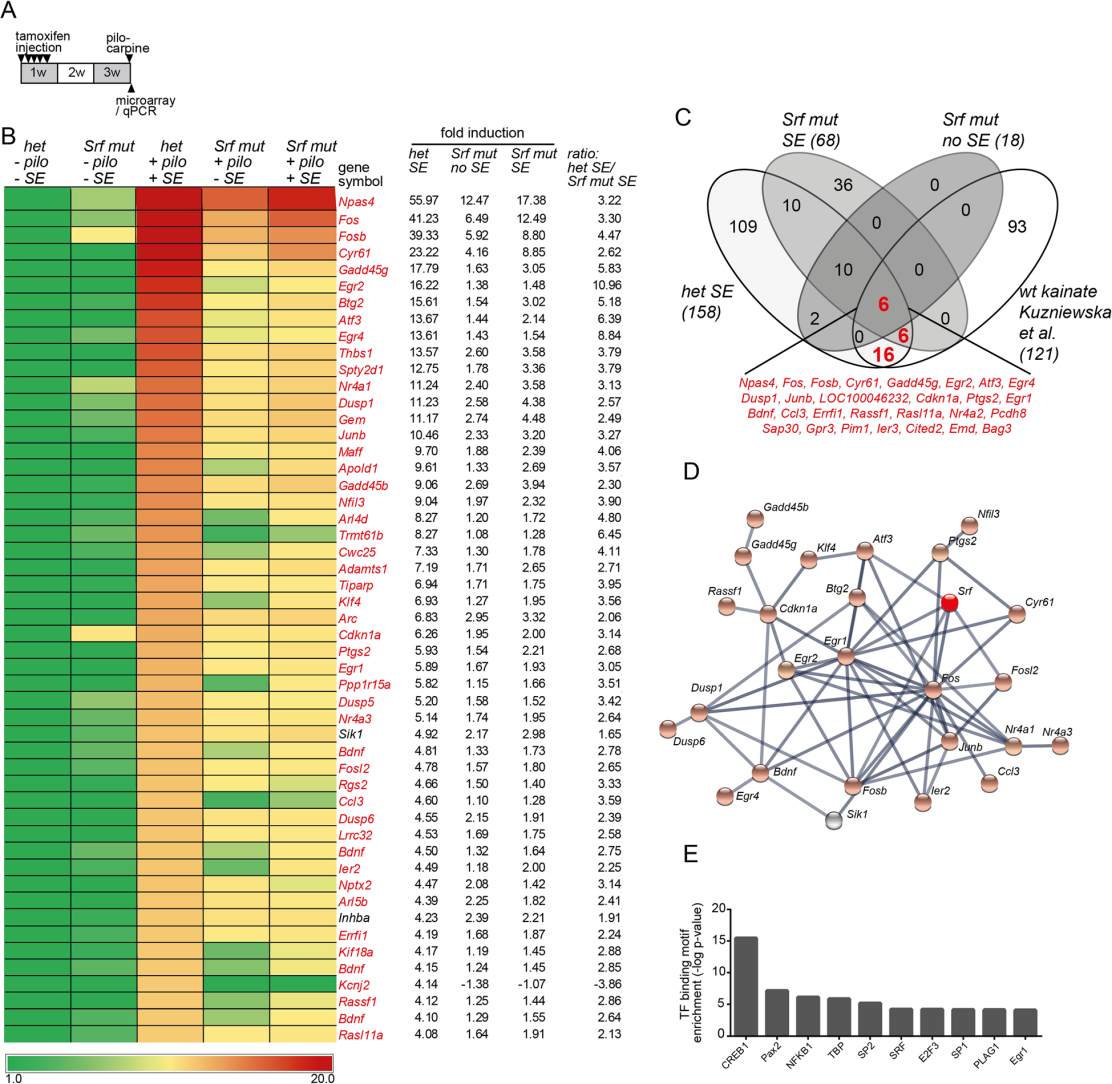
SE induced gene regulation requires SRF. **a** Microarray experiments were performed with hippocampal samples, harvested in heterozygous and *Srf* mutant mice 40 min after SE or without SE. **b** Heat-map of the 55 top-regulated genes with at least a four-fold induction when comparing heterozygous mice with and without SE. We used *Srf* mutant mice injected with pilocarpine (“+pilo”), either not reaching (“-SE”) or reaching SE (“+SE”) status. Here, pilocarpine induced gene regulation was clearly diminished as indicated by reduced ratio of fold induction between heterozygous SE and *Srf* mut SE. Those genes affected by SRF deficiency at least two-fold are highlighted in *red*. **c** All genes induced more than two-fold in the four data sets indicated were subjected to Venn diagram analysis. This identified a core gene set comprised of 28 genes (labeled in *red*) upregulated in mice with either a pilocarpine or kainic acid induced SE. **d** STRING analysis of the 55 top-regulated genes in heterozygous mice with SE (see **b**) and *Srf*. Many genes have reported interactions with each other and *Egr1* and *Fos* emerged as nodes in this STRING network. Genes depicted in *light red* were regulated in an SRF dependent manner. **e** Overrepresentation analysis of transcription factor binding sites was performed with the 55 top-regulated genes (see **b**). Amongst the ten most strongly predicted TFs, CREB1, SRF and its downstream effector Egr1 were found

Seizures induce neuronal gene expression encompassing rapid upregulation of IEGs such as Fos and Egr family members [22]. Gain- and loss-of function studies with several stimuli have shown a role of SRF in IEG induction before [21]. Next, we addressed whether pilocarpine induced gene expression also requires SRF (Fig. 2).

For this, we employed genome-wide transcriptomic analysis in the hippocampus with five experimental groups (Fig. 2 and Additional file 1: Table S1). This included heterozygous and *Srf* mutant mice without pilocarpine administration (−pilo, −SE; *N* = 3 animals each). Furthermore, we used pilocarpine injected *Srf* mutant mice with grade 4 seizures but not reaching SE (+ pilo, −SE; *N* = 3). Since the absence of an SE might affect the strength of the gene response in *Srf* mutant mice, we included also *Srf* mutant mice reaching SE status for 40 min (+ pilo, + SE; *N* = 2). Finally, we employed heterozygous mice with a pilocarpine induced SE lasting 40 min (+ pilo, + SE; *N* = 3).

In control mice experiencing an SE, 50 genes were induced more than four-fold (depicted in the heat-map in Fig. 2b) and 208 genes were upregulated two-fold or more (Additional file 1: Table S1). In contrast to gene induction, there was less gene downregulation during SE. Here, only five genes were more than four-fold downregulated in heterozygous mice (Additional file 1: Table S1). Non-epileptic heterozygous and *Srf* mutant mice differed only in seven genes altered by a factor of 1.5 or more. These included actin encoding genes such as *Actl8* and *Actb* (Additional file 1: Table S1).

In heterozygous control mice injected with pilocarpine, the top induced genes included several IEGs such as *Fos, Fosb, Cyr61, Egr2* and *Egr4*. In addition, other genes such as *Npas4*, *Gadd45g* and e.g. *Atf3* with IEG like properties were also strongly induced by pilocarpine in control mice (Fig. 2b). In addition, several *Dusp* family members *(Dusp1, Dusp5* and *Dusp6*), inhibitors of MAP kinase signaling were also induced in heterozygous mice after SE (Fig. 2b). In general, the Gene Ontology (GO) term “MAP kinase signaling pathway” was significantly over-represented by 13 genes (*Fos, Jun, Junb, Rasgrf1, Bdnf, Dusp1, Dusp4, Dusp5, Dusp6, Gadd45b, Gadd45g, Il1a, Nr4a1*) in epileptic control mice (Additional file 1: Table S1). Many of these genes were affected by SRF ablation (Fig. 2b) suggesting an impact of SRF on MAP kinase signaling to be further analyzed in this study.

In the hippocampus of *Srf* mutant mice with seizure activity (stage 4 to 5), however without an SE period, pilocarpine failed to induce gene expression to the same extent. Now, essentially all 50 genes induced strongly in heterozygous mice (>4 fold) were only weakly or not at all induced in the absence of SRF (Fig. 2b). Since stage 4 to 5 seizures might not result in the same extent of gene induction as an SE, we also analyzed *Srf* mutant mice experiencing 40 min of SE. As shown before (Fig. 1), these *Srf* mutant mice show a comparable SE intensity compared to heterozygous mice. Here, gene expression induced by pilocarpine was at least two-fold reduced in 49 out of 50 top-regulated genes when compared to epileptic control mice (Fig. 2b). This supports a role of SRF in pilocarpine induced gene expression.

A recent study showed SRF dependent gene expression in the kainic acid model after a 6 h timepoint in the hippocampal dentate gyrus [22]. We used Venn diagrams to decipher shared genes induced in both, the pilocarpine (Fig. 2b) and kainic acid [22] epilepsy model (Fig. 2c). Interestingly, in control mice, pilocarpine and kainic acid shared a gene set composed of 28 genes including several IEGs such as *Fos, Fosb, Cyr61* and *Egr* family members (labeled in red in Fig. 2c). These 28 genes account for approximately 20–25% of the total top-upregulated genes in either epilepsy model.

For the pilocarpine induced gene network of heterozygous control mice we performed STRING analysis including the 50 top-regulated genes with ≥4-fold induction and *Srf* itself (Fig. 2d). Approximately half of these genes were connected with each other at least by one interaction. Within this gene network, the SRF target genes *Fos* and *Egr1* emerged as hubs connected to many upregulated genes.

Data obtained so far point at a role of SRF in pilocarpine mediated gene induction. This was supported by promoter analysis of all genes with at least four-fold induction (Fig. 2e). Here, SRF and Egr1 were identified amongst the ten transcription factors predicted to have highest numbers of binding motifs in these pilocarpine-induced genes (Fig. 2e). CREB1 was the top-ranked transcription factor identified by this algorithm (Fig. 2e). So far, CREB is considered a prototype TF to regulate neuronal activity mediated gene expression [19]. Nevertheless, data by Kuzniewska [22] together with ours (Fig. 2b) suggest no major functional compensation of seizure-induced gene expression by CREB upon SRF ablation.

**Fig. 3:**
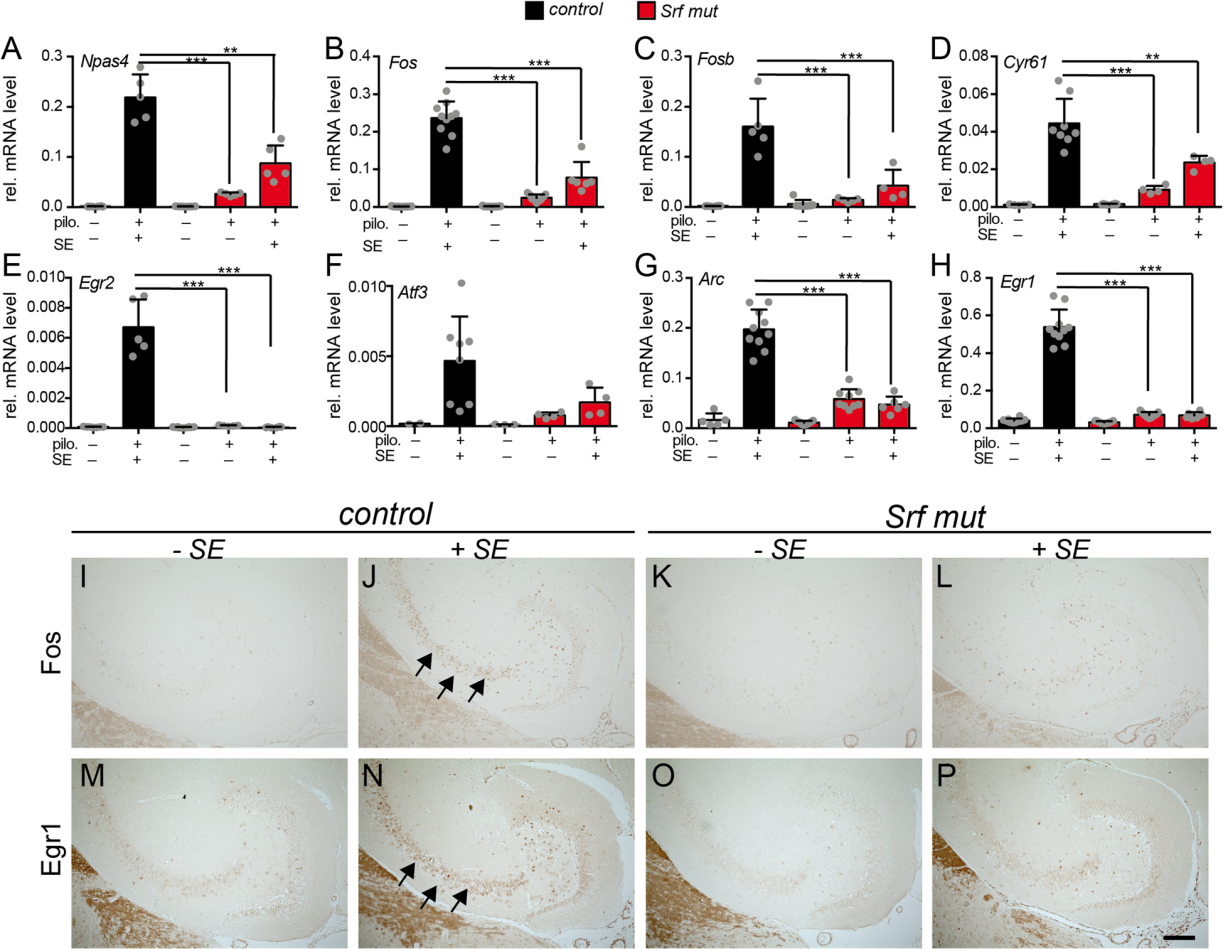
Validation of SRF mediated gene regulation during SE. **a**-**h** qPCR validation of SE microarray results with cDNAs derived from independent hippocampal samples. Pilocarpine injected (+pilo) *Srf* mutant animals either reached SE (+SE) or not (−SE). In heterozygous mice with SE, all IEGs, *Fos* (**b**), *Fosb* (**c**), *Cyr61* (**d**), *Egr2* (**e**), *Arc* (**g**) and *Egr1* (**h**) were upregulated during a 40 min SE, whereas this was significantly reduced in SRF deficient mice with SE. The same holds true for two other genes, *Npas4* (**a**) and *Atf3* (**f**). Data are represented as mean ± SD. Individual animals are labeled with *grey circles*. **i**-**p** Hippocampal sections were labeled with Fos (**i**-**l**) or Egr1 (**m**-**p**) directed antibodies. In the absence of an SE, neither Fos (**i**, **k**) nor Egr1 (**m**, **o**) were present in heterozygous (**i**, **m**) or *Srf* mutant (**k**, **o**) hippocampal tissues. In control animals with an SE, Fos (**j**) and Egr1 (**n**) were upregulated in the CA3 region (see *arrows* in **j**, **n**) and dentate gyrus (DG) on the protein level, whereas this was less pronounced in SRF deficient animals (**l**, **p**). *Scale-bar* (**i**-**p**) = 100 μm

Finally, we corroborated SRF dependent target gene regulation by qPCR with independent cDNA samples (Fig. 3). Here, hippocampal samples or sections of heterozygous and *Srf* mutant mice experiencing an SE period for 40 min were analyzed (≥ *N* = 4; each condition). Indeed, microarray data for eight SRF dependent target genes predominantly encoding for IEGs such as *Fos, Fosb* or *Egr1* were confirmed by qPCR (Fig. 3a-h). In addition, we identified potentially new SRF target genes during pilocarpine-induced seizures such as the neuronal activity regulated transcription factor *Npas4* [59] and the regeneration associated gene (RAG) *Atf3* (Fig. 3a, f). Besides transcript level (Figs. 2 and 3), we confirmed microarray and qPCR data on protein level (Fig. 3i-p). For this, hippocampal sections of heterozygous and SRF deficient mice without an SE or with 40 min in SE were stained with Fos (Fig. 3i-l) or Egr1 (Fig. 3m-p) directed antibodies. In the absence of epileptic activity, Fos (Fig. 3i, k) and Egr1 (Fig. 3m, o) were barely detectable in heterozygous (Fig. 3i, m) or SRF deficient (Fig. 3k, o) tissue. After an SE, Fos and Egr1 were upregulated in control mice (Fig. 3j, n) but not as pronounced in SRF deficient animals (Fig. 3l, p).

In summary, gene expression evoked by pilocarpine-induced seizures required SRF.

### SRF ablation enhances spontaneous recurrent seizure (SRS) frequency

**Fig. 4:**
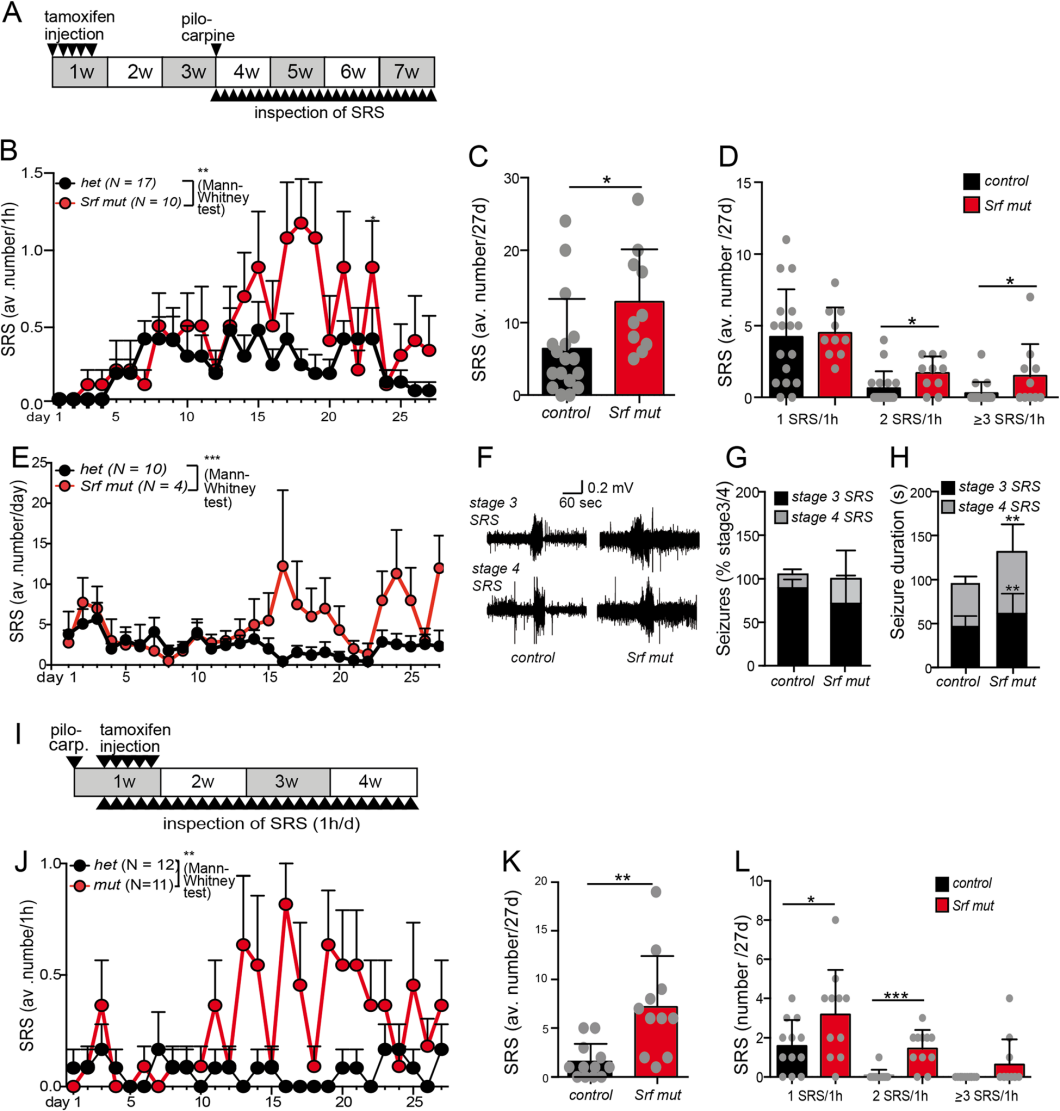
Spontaneous seizure frequency is elevated in SRF deficient mice. **a** Mice were tamoxifen injected and 14 days later SE was induced with pilocarpine. Subsequently, SRS occurrence was quantified by visual inspection of cages for 1 h/day (**b**-**d**) over 27 days. **b** Compared to heterozygous mice (*black line*), SRF deficient mice (*red line*) had significantly more SRS at several days throughout the 27 days observation period. **c** The total SRS number was doubled in *Srf* mutant compared to heterozygous mice over the 27 days. **d** In the daily 1 h observation period, there were significantly more SRF deficient mice with two (2 SRS/1 h) or three and more (≥3 SRS/1 h) SRS compared to heterozygous mice. **e** Similar to visual inspection (**b**), EEG recordings over 24 h/day for 27 days showed increased SRS numbers in SRF deficient mice. **f** Typical EEG traces of a stage 4 and 5 SRS in a control and *Srf* mutant animal. No obvious changes in SRS intensity were observed. **g** Heterozygous and *Srf* mutant animals had a comparable frequency of stage 4 and stage 5 SRS. **h** Upon SRF ablation, single SRS duration was significantly longer than in control animals. **i** In experiments to panels (J-L), the order of tamoxifen and pilocarpine injection was reversed compared to (**b**-**h**). After pilocarpine injection, SRS occurrence was observed for 1 h/day for 27 days. **j** SRF deficient mice had significantly more SRS at several days in the observation period. **k** The total SRS number/27 days was three- to four-fold elevated upon SRF ablation. **l** SRF deficiency resulted in more mice experiencing two or three and more SRS within the 1 h observation period. Data are represented as mean ± SD. Number of animals are indicated or individual animals are labeled with *grey circles*

So far, we analyzed the influence of SRF deficiency on the initial SE phase in the pilocarpine model (Figs. 1 and 2). Since this initial SE period triggers spontaneous seizures (SRS) in subsequent weeks, we analyzed SRF’s role in SRS occurrence (Fig. 4).

After pilocarpine injection we analyzed SRS occurrence in the home cages of heterozygous (*N* = 17) and *Srf* mutant mice (*N* = 10) by daily observation for 1 h over 4 weeks (Fig. 4b-d). To ensure that mice were in a comparable phase of their circadian rhythm, the daily 1 h observation period was always between 12.00 and 13.00. In the first week after SE, SRS number was low, regardless of genotype. After 7 days, SRS frequency in heterozygous control mice increased and remained constant thereafter for the 25d observation period (Fig. 4b). In *Srf* mutant mice, SRS number was more than doubled compared to heterozygous control mice (Fig. 4b). Thus, between two to 4 weeks after SE, each *Srf* mutant mouse analyzed experienced one SRS in the 1 h observation period (Fig. 4b). The total SRS number summed up over the 27 days observation period was two-fold elevated in *Srf* mutant compared to heterozygous mice (Fig. 4c). We noted that many *Srf* mutant mice not only experienced one SRS during the 1 h inspection, but rather two or more SRS were observed. Indeed, the number of SRF deficient mice experiencing either two (2 SRS/1 h) and three or more (≥3 SRS/1 h) SRS was significantly increased compared to heterozygous mice (Fig. 4d).

Besides visual inspection of home cages (Fig. 4b-d), SRS occurrence was quantified by combined EEG/video recording (Fig. 4e-h). Similar to visual quantification of SRS frequency, 24 h EEG recording over 27 days demonstrated significantly more SRS numbers upon SRF depletion (Fig. 4e). In contrast to SRS numbers, the EEG intensity during single SRS periods was unchanged between heterozygous and *Srf* mutant mice (Fig. 4f) as was the frequency of stage 4 or stage 5 SRS stages (Fig. 4g). However, the average duration of individual stage 4 or stage 5 SRS was significantly expanded by 20–30 s upon SRF ablation (Fig. 4h).

Previously we analyzed mice with SRF being deleted before pilocarpine mediated SE induction (Fig. 4a-h). In order to corroborate SRF’s function during SRS independent of the initial SE phase we reversed the order of tamoxifen and pilocarpine administration (Fig. 4i). Now, mice carrying one or two floxed *Srf* alleles were injected with pilocarpine first to elicit SE activity regardless of genotype. Subsequently, tamoxifen was injected to induce Cre-ERT2 mediated *Srf* recombination and mice were observed for SRS occurrence daily for 1 h over 4 weeks (Fig. 4j). As observed above (Fig. 4b, e), SRF deficient mice showed significantly elevated SRS numbers during many days, particularly between 2 and 4 weeks of observation (Fig. 4j). The average SRS number in the total observation period (27d) was more than three-fold elevated in mutant compared to control mice (Fig. 4k). Furthermore, the number of *Srf* mutant mice with two SRS/1 h was significantly increased compared to heterozygous mice (Fig. 4l).

Taken together, we show elevated SRS numbers upon SRF ablation in the pilocarpine model.

### SRF is required for SRS induced gene expression

Gene expression responses during the SE period require SRF [22]. Besides the SE stage, alterations for specific genes such as *Fos* were reported during an SRS in the chronic epilepsy phase [60]. However, to the best of our knowledge, so far no genome-wide mRNA expression profiling is available investigating gene expression changes immediately after an SRS in any mouse epilepsy model.

**Fig. 5:**
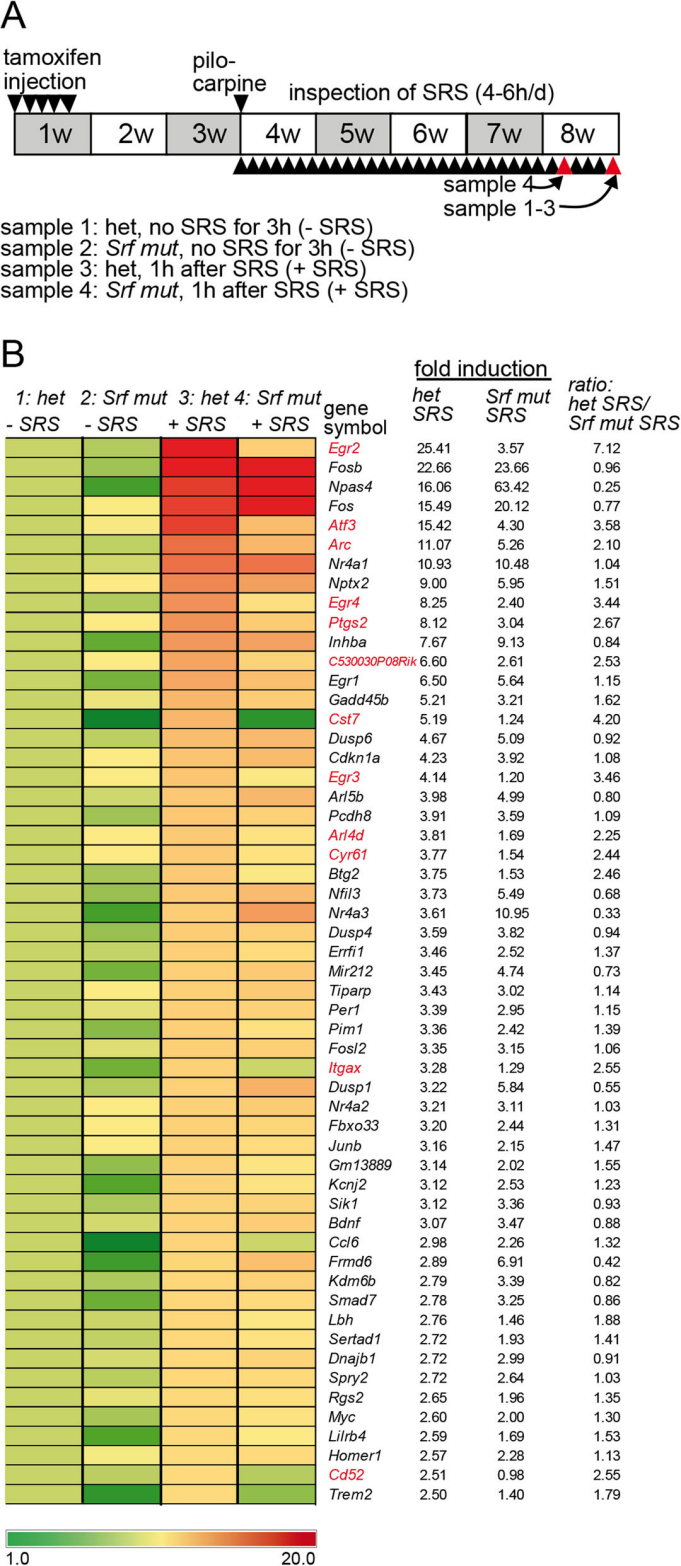
Spontaneous seizures elicit a gene response in an SRF dependent manner. **a** After pilocarpine injection, mice were observed for 4 to 6 h per day to collect samples for microarray analysis. *Red triangles* indicate days at which samples were collected. For SRS samples (+SRS), mice had to be free of an SRS for 3 h, followed by an SRS and hippocampus dissection 1 h later. For control samples, mice were in a chronic epileptic phase, however were free of an SRS for 3 h (−SRS). **b** Heat-map of all genes upregulated in SRS positive heterozygous mice with ≥2.5 fold induction. Genes depicted in *red* were affected by SRF deficiency with at least a two-fold change in the fold-induction ratio between control and mutant

In this study, we provide such a first SRS dependent transcriptome analyses (Fig. 5). For this, heterozygous and *Srf* mutant mice with an SE were observed daily for 4-6 h over 28 days. To obtain SRS specific samples, mice had to be SRS free for at least 3 h, followed by experiencing a single SRS phase lasting 30–60 s. One hour after the SRS, hippocampi were subjected to microarray profiling (“+SRS”, 1 h after SRS; Fig. 5a). As control, hippocampi of animals with an SE were included which regularly experienced SRS, however not within 3 h before tissue preparation (“-SRS”, no SRS for 3 h; Fig. 5a). In the days before harvesting hippocampal tissue for microarray analysis (see scheme in Fig. 5a), the four mice used experienced the following total SRS numbers: het/−SRS (2 SRS), het/+SRS (5 SRS), *Srf* mut/−SRS (10 SRS), *Srf* mut/+SRS (11 SRS).

In heterozygous mice, 105 and 13 genes were ≥ two-fold up- or downregulated, respectively. Thus, eight-fold more genes were induced by an SRS than down-modulated. In hippocampi of control mice, an SRS induced several IEGs such as *Egr2, Fosb, Fos* and e.g. *Arc* (Fig. 5b). Besides these, several other genes such as Dusp (*Dusp6, Dusp4 and Dusp1*) and nuclear receptor family members (*Nr4a1, Nr4a3, Nr4a2*), *Npas4* and *Atf3* were upregulated by an SRS (Fig. 5b). Of note, mRNA induction was in general weaker in SRS compared to SE samples (compare Figs. 2b and 5b). Next, we inspected the requirement of SRF for SRS mediated gene induction. In *Srf* mutant mice with an SRS, approximately 25% of the top 50 induced genes (13/55) were at least two-fold reduced compared to heterozygous mice (labeled in red in Fig. 5b). Thus, in contrast to SE with almost 100% of genes being regulated in an SRF-dependent manner (see Fig. 2b), SRS induced gene expression requires SRF to some extent but also other gene regulators.

**Fig. 6:**
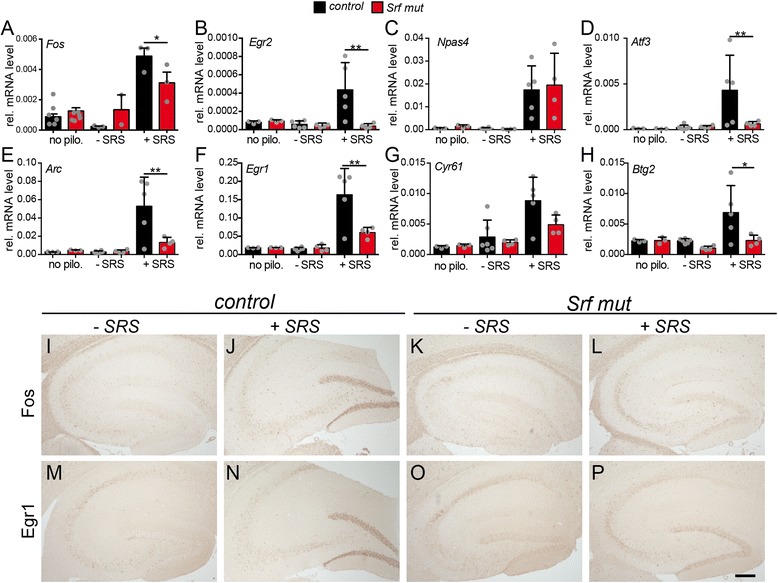
Validation of SRF mediated gene regulation during SRS. **a**-**h** qPCR validation of SRS microarray results with cDNAs derived from independent hippocampal samples. Experiments included animals with an SRS (+SRS) and, as control, we included mice with no pilocarpine injection (no pilo.) and epileptic mice not experiencing an SRS within the last 3 h (−SRS). The last two samples showed almost identical mRNA levels for all genes tested. In contrast, 1 h after an SRS, *Fos* (**a**), *Egr2* (**b**), *Atf3* (**d**), *Arc* (**e**), *Egr1* (**f**), *Cyr61* (**g**) and *Btg2* (**h**) were induced in heterozygous mice. This induction required SRF activity as shown by reduced mRNA levels in *Srf* mutant animals. In contrast, *Npas4* (**c**) was induced by an SRS, however not in an SRF dependent manner. Data are represented as mean ± SD. Individual animals are labeled with *grey circles*. For clarity, statistical significance is only depicted for conditions –SRS and +SRS of heterozygous and *Srf* mut mice. **i**-**p** Hippocampal sections of control mice and mice with an SRS were labeled with Fos (**i**-**l**) or Egr1 (**m**-**p**) directed antibodies. In the absence of an SRS, only weak Fos (**i**, **k**) or Egr1 (**m**, **o**) expression was present in heterozygous (**i**, **m**) or *Srf* mutant (**k**, **o**) hippocampi. In heterozygous animals with an SRS, Fos (**j**) and Egr1 (**n**) were upregulated in the hippocampal dentate gyrus, whereas this was decreased in SRF deficient animals (**l**, **p**). *Scale-bar* (**i**-**p**) = 100 μm

Finally, microarray data were confirmed by qPCR analysis of independent cDNA samples (Fig. 6). Here, we included hippocampi of heterozygous and *Srf* mutant animals with an SRS 1 h before (+SRS) or no SRS (−SRS) within 3 h (see above). In order to provide sufficient replicates for statistical testing, at least four animals were analyzed for each experimental condition (*N* = 4; Fig. 6). Additionally, we included heterozygous and mutant animals with no SE to compare mRNA abundance between animals not in a chronic epilepsy phase (no pilo.) and in a chronic epilepsy phase. No major differences were observed between “–SRS” and “no pilo.” samples, suggesting that epileptic control animals with no acute SRS do not induce IEGs (Fig. 5b). All IEGs, *Fos* (Fig. 6a), *Egr2* (Fig. 6b), *Arc* (Fig. 6e), *Egr1* (Fig. 6f) and *Cyr61* (Fig. 6g) were upregulated by an SRS in heterozygous mice. This induction was significantly reduced upon SRF ablation as seen in microarray data, except for *Npas4* and *Cyr61* (Fig. 5b). In addition, *Atf3* (Fig. 6d) and *Btg2* (B cell translocation gene 2; Fig. 6h) induction was SRF dependent. In contrast, *Npas4* was elevated by SRS, however not depending on SRF (Fig. 6c).

SRS data on transcript level (Figs. 5 and 6a-h) were confirmed on protein level by Fos and Egr1 directed histology on hippocampal sections (Fig. 6i-p). In heterozygous mice an SRS resulted in Fos and Egr1 induction primarily in the hippocampal dentate gyrus (Fig. 6j, n). This induction was reduced in SRF deficient animals with an SRS (Fig. 6l, p).

In summary, we provide a first analysis of early changes in the SRS transcriptome and show a contribution of SRF in stimulating this gene response.

### SRF mediates seizure-induced neuron loss and mossy fiber sprouting

**Fig. 7:**
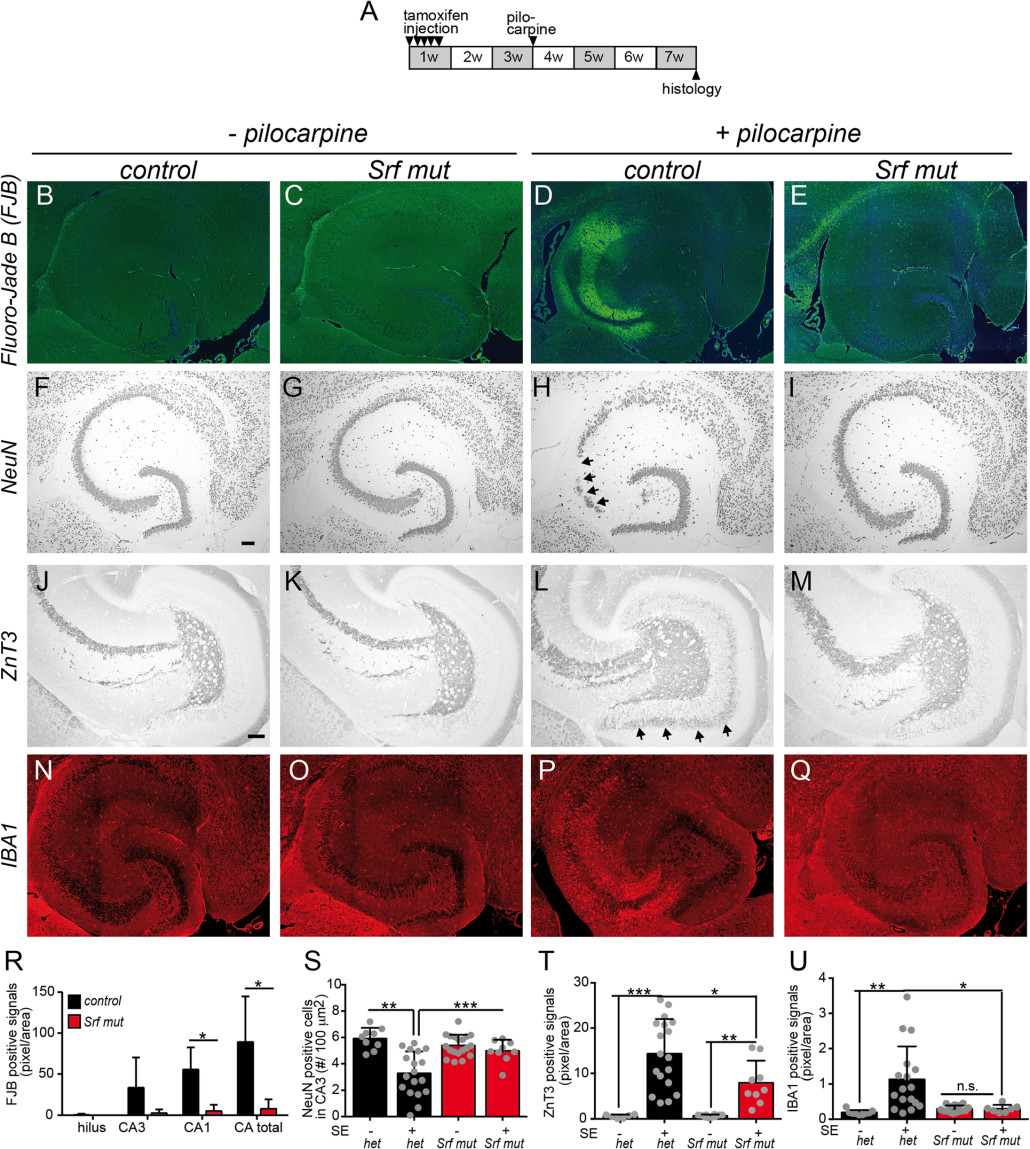
SRF ablation modulates epilepsy associated neuron loss, mossy fiber sprouting and inflammation. **a** Four weeks after induction of an pilocarpine-mediated SE, hippocampi of heterozygous and SRF deficient animals were analyzed histologically with several markers as described below. **b**-**e** Fluoro-Jade B (FJB) labels degenerating neurons. Expectedly, no FJB signals were detectable in non-epileptic heterozygous (**b**) or mutant (**c**) hippocampi. Four weeks after an SE, FJB signals were increased in heterozygous (**d**) but not SRF deficient (**e**) animals. **f**-**i** NeuN labels all neuronal nuclei. In epileptic control animals, neuron loss was observed in the CA3 region (*arrows* in **h**). This was diminished in mutant animals with an SE (**i**). **j**-**m** ZnT3 labels hippocampal mossy fibers. No mossy fiber sprouting by granule cells was observed in the molecular layer of the dentate gyrus of non-epileptic heterozygous (**j**) or mutant (**k**) animals. In contrast, mossy fiber axon sprouting was observed in heterozygous animals with an SE (*arrows* in **l**) but not as pronounced in *Srf* mutant animals (**m**). **n**-**q** Microglia were labeled by anti-IBA1 directed antibodies. Only in epileptic control animals (**p**), a strong presence of microglia was observed. Of note, the area occupied by microglia was identical to the region of strongest neuronal demise (compare **p** with **d**). **r**-**u** Quantification of FJB (**r**), NeuN (**s**), ZnT3 (**t**) and IBA1 (**u**) signals in the hippocampus. Data are represented as mean ± SD. Individual animals are labeled with *grey circles*. *Scale-bar* (**b**-**i**; **n**-**q**) = 200 μm; (**j**-**m**) = 75 μm

In human TLE, seizures evoke neuron loss and hippocampal moss fiber sprouting, however the cause/effect relationship between seizures and anatomical alterations is not completely resolved ([9, 10]; see discussion). The pilocarpine model recapitulates such seizure-associated processes [12]. So far, the contribution of transcription factors to these processes has not been analyzed in great detail in any mouse epilepsy model. Since mossy fiber sprouting requires cytoskeletal dynamics and SRF particularly impinges on actin-based cytoskeletal alterations, SRF’s contribution to these processes was analyzed next (Fig. 7).

For this, heterozygous animals acquired a pilocarpine-induced SE period. In addition, we included *Srf* mutant animals which experienced an SE comparable to heterozygous mice (*N* ≥ 9, each condition). Four weeks later, histological analysis with the neurodegeneration marker Fluoro-Jade B (FJB), the pan-neuron marker NeuN, the mossy fiber marker ZnT3 and anti IBA1 directed antibodies to label microglia were performed in the hippocampus (Fig. 7a).

In non-epileptic heterozygous or *Srf* mutant animals, expectedly no FJB signals were visible (Fig. 7b, c). Four weeks after pilocarpine injection, FJB signals in the hippocampus of control animals, indicative of neurodegeneration, were readily visible (Fig. 7d; quantified in R). In contrast to this, *Srf* mutant mice with SE and subsequent chronic seizure phase showed strongly reduced FJB signals (Fig. 7e; R). In agreement, quantification of NeuN positive neurons confirmed SE-mediated neuron loss in epileptic heterozygous animals, particularly in the CA3 region (arrows Fig. 7h; quantified in S). Once again, in epileptic *Srf* mutant animals CA3 neuron loss was diminished (Fig. 7i; quantified in S).

In the absence of seizures, the mossy fiber projection labeled with ZnT3 of heterozygous and SRF deficient animals was identical (Fig. 7j, k). In epileptic heterozygous mice, mossy fiber sprouting was strongly induced (arrows Fig. 7l; quantified in T). Upon SRF ablation, epileptic mice failed to induce mossy fiber sprouting to the same extent as seen in control mice (Fig. 7m; T). Thus, in *Srf* mutant mice decreased mossy fiber sprouting correlated with enhanced spontaneous seizure frequency.

Epilepsy is associated with an inflammatory response of brain resident immune cells such as astrocytes and microglia [61]. We investigated seizure-mediated inflammation by analyzing IBA1 positive microglia (Fig. 7n-q; U) and GFAP positive astrocytes (data not shown). Epileptic seizures enhanced microglia numbers in heterozygous control animals (Fig. 7p) but less in SRF deficient animals (Fig. 7q). The area occupied by microglia in the CA3 region was identical to the strongest neuron loss identified by NeuN (compare Fig. 7p with H). Similar data were obtained for astrocytes, although statistical significance was not reached (data not shown).

Finally, we analyzed an interaction of epilepsy with neurogenesis, an important factor modulating brain plasticity and cognitive comorbidities in TLE patients [62]. For this, hippocampal sections of heterozygous and SRF deficient animals were labeled with doublecortin (DCX), a marker of newly generated neurons in the dentate gyrus (Additional file 2: Figure S2). DCX positive neurons were elevated in non-epileptic SRF deficient animals compared to control mice suggesting enhanced neurogenesis upon SRF ablation (Additional file 2: Figure S2). Since ablation of DCX positive neurons reduces SRS frequency [63], elevated numbers of DCX positive neurons might contribute to an increased SRS frequency, as observed upon SRF ablation.

In summary, SRF depletion reduced neurodegeneration, mossy fiber sprouting and inflammation.

### SRF directly regulates *Dusp* family members during epilepsy

**Fig. 8:**
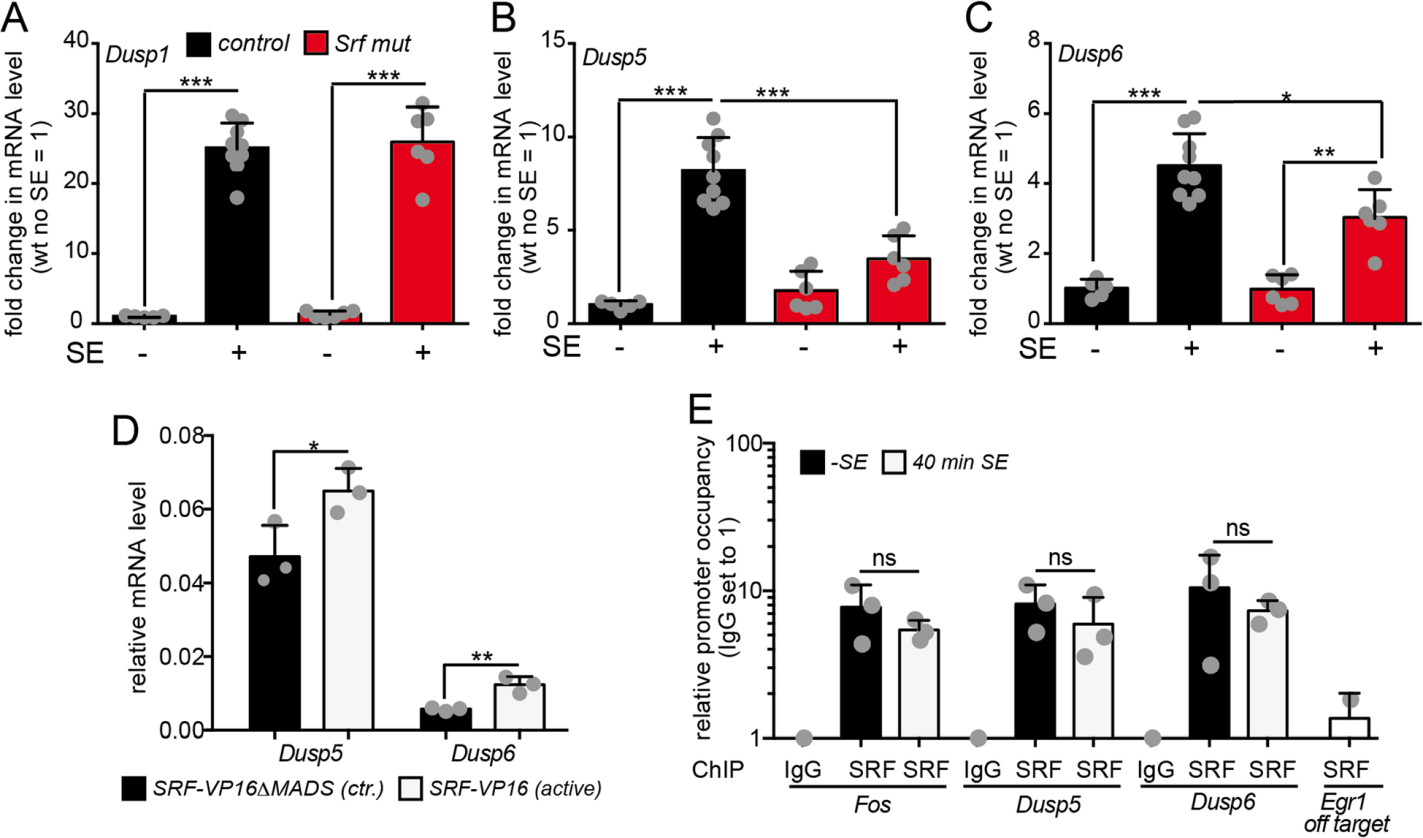
SRF regulates abundance of *Dusp* family members. **a**-**c** mRNA levels of *Dusp1* (**a**), *Dusp5* (**b**) and *Dusp6* (**c**) in the hippocampus of heterozygous and *Srf* mutant animals without or with 40 min SE was quantified by qPCR. *Dusp1* was upregulated during SE irrespective of genotype (**a**). *Dusp5* (B) and *Dusp6* (**c**) were induced by an SE in control mice but not as strongly upon SRF ablation. **d** Primary neurons overexpressed an SRF control protein (SRF-VP16ΔMADS) or constitutively-active SRF-VP16. SRF-VP16 upregulated mRNA abundance of both, *Dusp5* and *Dusp6*. **e** Hippocampal tissue of heterozygous animals without or with SE was subjected to SRF directed ChIP followed by qPCR for target genes indicated. SRF occupied the promoter of the well-established target gene *Fos.* SRF was also found at *Dusp5* and *Dusp6* promoters with comparable intensity to *Fos*. SRF occupancy was slightly reduced upon SE induction at all promoters. Only low SRF levels were found at an *Egr1* gene position not harboring SRF binding sites (*Egr1* off target). As further control, unspecific IgG antibodies were employed resulting only in low qPCR signals. Data are represented as mean ± SD. Individual animals are labeled with *grey circles*

In microarrays we observed induction of several *Dusp* family members (i.e. *Dusp1, Dusp5* and *Dusp6*) after pilocarpine injection (Fig. 2). DUSP induction during epilepsy has been previously reported in patients [7, 64] as well as rodent models [22, 41, 42, 65, 66]. So far, no transcription factor has been identified regulating expression of these MAP kinase antagonists in neurons. We employed gain- and loss-of-function experiments as well as chromatin immunoprecipitation (ChIP) to demonstrate direct *Dusp* regulation by SRF (Fig. 8).

In the hippocampus of heterozygous and mutant mice with an SE, mRNA abundance of all three *Dusp* members, *Dusp1* (Fig. 8a), *Dusp5* (Fig. 8b) and *Dusp6* (Fig. 8c) was strongly induced by a 40 min SE period. Pilocarpine mediated induction of *Dusp5* and *Dusp6* required SRF, as revealed by reduced mRNA levels in SRF deficient mice (Fig. 8b, c). In opposite to this, *Dusp1* induction was SRF independent (Fig. 8a), in contrast to microarray data (Fig. 2).

We further analyzed *Dusp5* and *Dusp6* further in SRF gain-of-function experiments using a constitutively active SRF protein (SRF-VP16) expressed in primary neurons. Complementary to reduced *Dusp* expression in SRF loss-of-function (Fig. 8a-c), SRF-VP16 upregulated *Dusp5* and *Dusp6* mRNA levels without further exogenous stimulation of neurons (Fig. 8d).

Finally, we report direct SRF occupancy at SRF binding sites (CArG boxes) of *Dusp5* and *Dusp6* promoter regions in the hippocampus of non-epileptic and epileptic mice (Fig. 8e). In SRF directed ChIPs, SRF was bound at the *Fos* as well as *Dusp5* and *Dusp6* promoter with comparable intensity (Fig. 8e). Induction of an SE resulted in a slight decrease in promoter binding for all three genes, however not in a significant manner. Control experiments with unspecific antibodies (IgG) and gene regions without SRF binding site (*Egr1* off target) did not result in robust qPCR signals (Fig. 8e).

Overall these experiments support direct *Dusp* gene regulation and promoter binding of SRF.

### MAP kinase signaling is enhanced upon SRF ablation

**Fig. 9:**
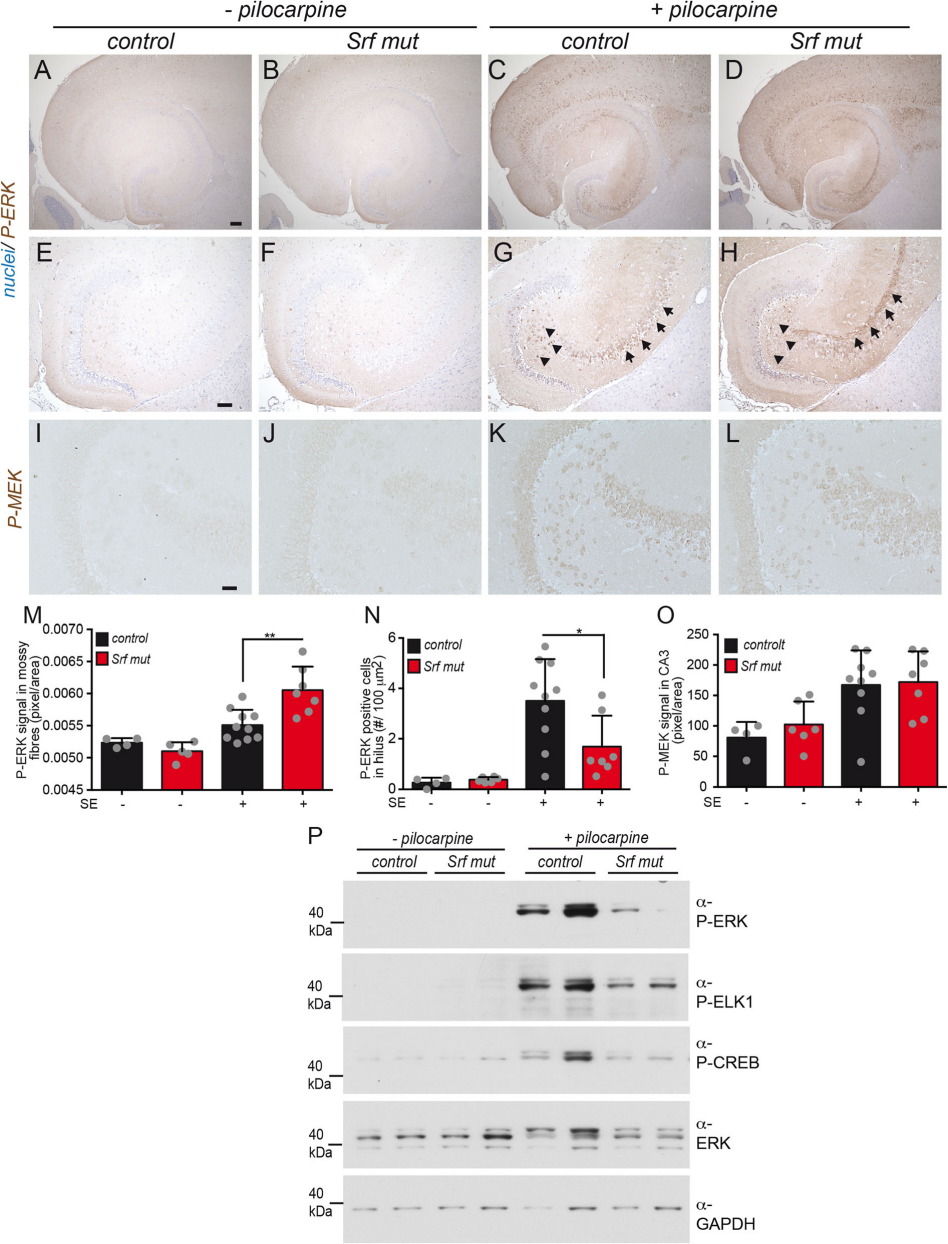
Activity of MAP kinase signaling members is altered upon SRF ablation. **a**-**h** Hippocampal sections were labeled with anti P-ERK directed antibodies and counterstained with Nissl. Without SE induction (−pilocarpine), almost no P-ERK signals were observed in heterozygous (**a**, **e**) or *Srf* mutant (**b**, **f**) animals. In heterozygous animals with SE, P-ERK levels in the hilus were upregulated (*arrowheads* in **g**) in contrast to *Srf* mutant animals (*arrowheads* in **h**). In addition, P-ERK levels were augmented in the CA3 region of epileptic control animals (**c**; *arrows* in **g**). This was even further increased upon SRF ablation and now P-ERK co-localized with mossy fibers (*arrows* in **h**). **e**-**h** are higher magnifications of (**a**-**d**). **i**-**l** P-MEK levels were low in heterozygous (**i**) and SRF deficient (**j**) animals without SE. Pilocarpine injection induced P-MEK in a similar manner in heterozygous (**k**) and mutant (**l**) animals. **m**-**o** Quantification of P-ERK levels in mossy fibers (**m**) and hilus (**n**) as well as of P-MEK (**o**). Data are represented as mean ± SD. Individual animals are labeled with *grey circles*. **p** Immunoblotting of heterozygous and SRF-deficient hippocampal lysates derived from animals without (−pilocarpine) or with 40 min SE (+pilocarpine). For each condition, two animals are depicted. P-ELK1, P-ERK and P-CREB levels were elevated by an SE in heterozygous control animals but not as much in *Srf* mutant animals. *Scale-bar* **a**-**d** = 200 μm; **e**-**h** = 75 μm; **i**-**l** = 100 μm

MAP kinase signaling is an important signaling pathway in epilepsy (see introduction). Since we observed regulation of *Dusp* phosphatases by SRF (Fig. 8), we analyzed MAP kinase activation in heterozygous and mutant epileptic mice (Fig. 9). For this, activation of ERK (i.e. phosphorylated ERK, P-ERK), the SRF cofactor ELK-1 (P-ELK-1) and of CREB (P-CREB) was investigated.

In mice without pilocarpine injection only weak P-ERK signals were observed in the hippocampus of both genotypes (Fig. 9a–f). Heterozygous mice experiencing an SE for 40 min strongly elevated P-ERK abundance in the cortex and hippocampus (Fig. 9c, g). In the hippocampus, these P-ERK signals were mainly observed in neurons residing in the hilus (arrowheads in Fig. 9g) and the CA3 region (arrows in Fig. 9g). Interestingly, when inspecting SRF deficient mice, we noted hippocampal sub-regions with elevated and decreased P-ERK signals compared to control mice (Fig. 9d, h). In the hilus, P-ERK abundance was significantly decreased compared to control mice (arrowheads Fig. 9h; quantified in N). In contrast, in the CA3 region we observed elevated P-ERK signals upon SRF ablation co-localizing with mossy fiber axons (arrows in Fig. 9h; quantified in M). This suggests a hippocampal region or neuron-specific modulation of MAP kinase signaling by SRF. The upstream activator of ERK, MEK, was likewise activated by epilepsy (Fig. 9k, l). However, we did not observe alterations in P-MEK activation between genotypes (Fig. 9k, l; quantified in O).

We further analyzed MAP kinase signaling by focusing on the hippocampal DG region using immunoblotting (Fig. 9p). As described above (Fig. 9a-h), P-ERK levels were elevated in epileptic heterozygous mice but not as much in *Srf* mutant animals (Fig. 9p). Since ERK phosphorylates and thereby activates ELK-1 and CREB in the nucleus, abundance of P-ELK-1 and P-CREB was analyzed. Both gene regulators were more strongly phosphorylated in pilocarpine treated mice of either genotype (Fig. 9p). However, similar to P-ERK, we observed enhanced P-ELK1 and P-CREB abundance in heterozygous compared to SRF depleted mice (Fig. 9p). Total CREB levels were unaltered (data not shown).

In summary, we observed modulation of MAP kinase activation and associated downstream transcription factors in SRF deficient mice.

## Discussion

### SRF’s function in epileptogenesis

So far, transcriptional regulation of SE acquisition is poorly understood. Herein, we uncovered a first SRF role in modulating SE acquisition using the pilocarpine model. Upon SRF ablation, mice showed reduced SE induction (Fig. 1). This result was obtained mainly by Racine scale scoring which does not allow for more quantitative assessment of SE strength (Fig. 1d). For this, we additionally employed EEG analysis (Fig. 1f-i). In those SRF deficient mice reaching an SE, all band power curves during EEG analysis were reduced compared to heterozygous control animals (Fig. 1h). However, none of the statistical testing (ANOVA, *t*-test) resulted in a statistical significance for any of the single power bands. Nevertheless, such a 50% reduction in band power observed in all bands in *Srf* mutant mice might have a biological impact on SE intensity (Fig. 1h). Thus, although we tried to include SRF deficient mice with comparable SE intensity as the control group in all experiments (as judged by the Racine scale), we cannot completely rule out a reduced SE intensity in *Srf* mutant mice. Thus, differences in e.g. pilocarpine induced gene expression, neurodegeneration and inflammation might partially be due to differences in the initial SE intensity between control and *Srf* mutant mice.

In contrast to pilocarpine, SRF ablation allowed for initial seizure acquisition in the kainic acid model [22]. This suggests an SRF dependence on the epilepsy inducing agent and associated signaling pathways. Thus, neuronal activity dependent signaling cascades via muscarinergic (pilocarpine) but not so much glutamate receptors (kainic acid) appear to target nuclear SRF function. Our data suggest that SRF modulates pilocarpine signaling not directly at the receptor level, since the M1 muscarinic receptor abundance was unchanged (data not shown) but further downstream e.g. at the MAP kinase level (see below).

Surprisingly*, Srf* mutants displayed more spontaneous seizures (SRS) despite the reduced SE occurrence suggesting a dual role of SRF in epileptogenesis (Fig. 4). So far, a similar phenotype has not been reported in other mouse mutants to the best of our knowledge. This elevated SRS frequency was not connected to the initial SE intensity as shown in mice with the SRF deletion being introduced after SE induction (Fig. 4i-l). In addition, our data are fully congruent with enhanced SRS numbers reported in the kainic acid model in *Srf* mutants [22]. Thus, regardless of the epilepsy-inducing agent, SRF emerges as important regulator of SRS activity.

So far, consequences of *Srf* deletion on epilepsy-associated neuropathology were not reported. We show decreased neurodegeneration, inflammation and mossy fiber sprouting in *Srf* mutant mice (Fig. 7). The extent of neurodegeneration and inflammation depends on SE intensity and duration. For instance, mice with seizure activity but no SE status or mice with shorter SE periods (<40 min employed in this study) had decreased neurodegeneration or inflammatory responses (data not shown). Therefore we only included animals experiencing the same SE duration (40 min) and SE intensity as scored by the Racine scale (Fig. 7).

Enhanced mossy fiber sprouting might be proepileptogenic by forming new excitatory seizure-generating circuits [67]. This is controversial since mossy fibers might also excite inhibitory interneurons thereby potentially decreasing seizure activity or might not affect SRS frequency at all [4, 68]. In SRF deficient mice we observed enhanced SRS numbers correlating with reduced mossy fiber sprouting (Fig. 7). Similarly, *Srf* mutant mice had decreased neuronal cell loss (Fig. 7) although they experienced more spontaneous seizures (Fig. 4). Thus, in epileptic wildtype mice SRF would reduce SRS activity and stimulate generation and growth of new mossy fiber sprouts formation, a function in line with reported SRF roles in promoting axon growth in the physiological brain [34–39]. Our findings favor a scenario in wt mice where these new axon sprouts form inhibitory rather than excitatory circuits, resulting in decreased SRS occurrence. In *Srf* mutant mice, mossy fiber sprout formation was reduced. This might result in failed inhibitory circuit formation and thereby contribute to the elevation of SRS numbers in *Srf* mutants. Although we do not provide experimental evidence for this, *Srf* ^*CaMKCreERT2*^ mice might be well suited to investigate such a potential antiepileptogenic function of mossy fiber sprouting in future studies.

### SRF’s function in seizure mediated gene expression

In this study we identified SRF as neuronal activity regulated transcription factor for SE and SRS associated gene expression in the pilocarpine model (Figs. 2, 3, 5, 6 and 8). For SRS elicited gene expression this is the first genome-wide screen so far reported (Fig. 5). We show that 1 h after a single SRS (lasting approx. 40 s; Fig. 4h) many IEGs were induced in an SRF dependent manner (Fig. 5). Interestingly, this gene set was significantly enriched for GO terms indicating an inflammatory response (data not shown, Additional file 1: Table S1). Thus, even short seizure episodes such as a single SRS might elicit inflammatory brain responses.

Taken together with data in the kainic acid model [22], SRF is involved in SE induced gene expression in two mouse models. Since in our study SRF ablation interfered with SE development (Fig. 1), reduced gene responses in these mice might be an indirect consequence of this dampened neuronal activation. However, to account for this we included in our experiments additionally *Srf* mutant mice reaching SE status (Figs. 2 and 3) and mutant mice with even more spontaneous seizures (Figs. 5 and 6). In both cases, gene expression was reduced upon SRF ablation arguing against decreased seizure strength as confounding factor.

The pilocarpine and kainic acid models shared a core set of 20–30 genes induced by SE accounting for 20–30% of the top-regulated SE genes (Fig. 2c). This conserved gene set mainly consists of typical IEGs such as *Fos, Fosb, Egr1, Egr2* and *Junb* (Fig. 2c) and was also induced in human TLE patients [7]. Given this overlap between mouse models and TLE patients, SRF might be an important regulator and potential future therapeutic target in human epilepsy. In opposite to this shared gene set, a majority of top-induced genes might be specific for pilocarpine and kainic acid signaling. However, also other factors diverging between the study of Kuzniewska and coworkers [22] and ours such as time-points and hippocampal region might account for this difference.

What might be the role of these IEGs as SRF effector genes during epilepsy? Many SRF regulated genes including *Fos, Fosb, Egr1, Egr2, Atf3* and *Npas4* encode for transcription factors. *Egr1* and *Fos* emerged as nodes within STRING networks and might directly or indirectly contribute to SRF target gene expression (Fig. 2d). Thus, in wildtype mice, SRF might mediate a first transcriptional response resulting in expression of TF encoding IEGs and these most likely initiate a second delayed gene expression wave resulting in expression of further effector genes. Notably, many single mouse mutants of e.g. *Fos* [69, 70], *Fosb* [71, 72], *Arc*, [73], *Npas4* [74], *Atf3* [75], and *Btg2* [75] but not of *Egr1* [76] show enhanced seizure activity, thus resembling epileptic *Srf* mutant mice. In *c-Fos* null mutants mossy fiber sprouting in the kindling model was reduced as observed upon SRF ablation [77]. Thus, although we do not provide functional data, it is conceivable that individual or combined IEG activities are important downstream SRF effectors.

### SRF modulates MAP kinase signaling during SE

We observed significant upregulation of the GO term “MAP kinase signaling” including *Fos, Jun, Junb, Rasgrf1, Bdnf, Dusp1, Dusp4, Dusp5, Dusp6, Gadd45b, Gadd45g, Il1a* and *Nr4a1*. Essentially all of these genes required SRF for full induction (Fig. 2). Since *Dusp* genes are upregulated during SE, we focused in further analysis on these effectors. We observed direct *Dusp5* and *Dusp6* regulation by SRF (Fig. 8), in agreement with non-neuronal cells [78]. DUSP phosphatases inhibit ERK kinases thereby providing negative feed-back regulation on MAP kinase signaling [79]. Given the reduced *Dusp* levels in epileptic SRF deficient animals, one might expect a concomitant rise in P-ERK. Indeed, P-ERK levels were elevated in selected areas in the hippocampus of SRF deficient animals (Fig. 9). P-ERK was increased in the mossy fibers but not in the corresponding cell bodies of mossy fibers, i.e. the granule cells of the dentate gyrus upon SRF ablation (Fig. 9e-h). The latter suggests that enhanced MAP kinase activation upon SRF ablation is spatially confined to the axons and not the cell bodies. Elevated MAP kinase signaling is reported to enhance SRS numbers [47]. This finding correlates with enhanced seizure occurrence upon SRF ablation observed in this study (Fig. 4) and by others [22]. Overall, one SRF function in epileptic wildtype mice appears to involve *Dusp* gene induction. Subsequently, although we do not show direct functional proof of such an SRF-MAP kinase signaling interaction, DUSP proteins might dampen MAP kinase activity and eventually reduce seizure occurrence. This regulatory loop appears to operate directly on ERK since upstream MAP kinase propagation at the MEK level was unaltered upon SRF ablation (Fig. 9).

Notably, although impaired *Dusp* gene induction suggests elevation of P-ERK levels upon SRF ablation, we also observed a region-restricted impairment of P-ERK activation upon SRF ablation in the hilus (Fig. 9). Several cell types are residing in the hilus including inhibitory interneurons. In *Srf* ^*CaMKCreERT2*^ mice, SRF ablation occurs in excitatory but not inhibitory interneurons suggesting an indirect effect of SRF on ERK activation in interneurons, e.g. through granule cell neurons synapsing with these interneurons. Reduced P-ERK signals in the hilus correlated with decreased levels of phosphorylated ELK-1 and CREB (Fig. 9). The SRF cofactor ELK-1 and CREB were previously reported to regulate neuronal IEG induction [31, 80, 81]. Thus, SRF might team-up with these two gene regulators for epilepsy mediated gene regulation.

Overall, we show first data on the role of SRF in MAP kinase signaling during epilepsy. Our data suggest, although very speculative at this stage that SRF might dampen or augment MAP kinase activity depending on the hippocampal sub-region (e.g. CA3 vs. hilus) or neuron type (e.g. excitatory vs. inhibitory neuron).

## Additional files

Additional file 1: Table S1. Excel sheet including all microarray data. (XLSX 44537 kb)

Additional file 2: Figure S1. SRF ablation does not interfere with entering a kainic acid induced seizure period. (A, B) Wt and *Srf* mutant animals were injected with 20 μg/g bodyweight kainic acid (Tocris). Subsequently, seizures in mice were scored according to the modified Racine scale for approximately 2 h. Both, wt (black line) and SRF deficient (red line) animals developed grade 3–5 seizures and we did not observe an obvious difference between genotypes (A). In addition, the latency until a first grade 3 seizure was not affected by SRF ablation (B). Data are represented as mean ± SD. Number of animals are indicated or individual animals are labeled with grey circles. **Figure S2.** SRF ablation elevates numbers of DCX positive neurons. (A, B) Wt (A) and SRF deficient (B) sections of the dentate gyrus were labeled with anti. Doublecortin (DCX) directed antibodies. DCX positive neurons were localized to the dentate gyrus subgranular zone (A, B). In Srf mutant animals, numbers of DCX positive neurons were elevated compared to wt. (C) Quantification of DCX positive neurons per μm of the dentate gyrus. Data are represented as mean ± SD. Individual animals are labeled with grey circles or black squares. Scale-bar (A, B) = 100 μm. (PDF 713 kb)

## Abbreviations

ChIP: Chromatin immunoprecipitation
GO: Gene ontology
Het: Heterozygous
IEG: Immediate early genes
SE: Status epilepticus
SRF: Serum response factor
SRS: Spontaenous recurrent seizure
TF: Transcription factor
TLE: Temporal lobe epilepsy

## References

[1] Strzelczyk A, Reese JP, Dodel R, Hamer HM (2008). Cost of epilepsy: a systematic review. PharmacoEconomics.

[2] Tellez-Zenteno JF, Hernandez-Ronquillo L (2012). A review of the epidemiology of temporal lobe epilepsy. Epilepsy Res Treat.

[3] Bozzi Y, Dunleavy M, Henshall DC (2011). Cell signaling underlying epileptic behavior. Front Behav Neurosci.

[4] Buckmaster PS (2014). Does mossy fiber sprouting give rise to the epileptic state?. Adv Exp Med Biol.

[5] Houser CR (1992). Morphological changes in the dentate gyrus in human temporal lobe epilepsy. Epilepsy Res Suppl.

[6] Houser CR, Zhang N, Peng Z, Huang CS, Cetina Y (2012). Neuroanatomical clues to altered neuronal activity in epilepsy: from ultrastructure to signaling pathways of dentate granule cells. Epilepsia.

[7] Beaumont TL, Yao B, Shah A, Kapatos G, Loeb JA (2012). Layer-specific CREB target gene induction in human neocortical epilepsy. J Neurosci.

[8] Rakhade SN, Yao B, Ahmed S, Asano E, Beaumont TL, Shah AK, Draghici S, Krauss R, Chugani HT, Sood S, Loeb JA (2005). A common pattern of persistent gene activation in human neocortical epileptic foci. Ann Neurol.

[9] de Lanerolle NC, Lee TS, Spencer DD: Histopathology of Human Epilepsy. In *Jasper's Basic Mechanisms of the Epilepsies.* 4th edition. Edited by Noebels JL, Avoli M, Rogawski MA, Olsen RW, Delgado-Escueta AV. Bethesda (MD); 2012.

[10] Sendrowski K, Sobaniec W (2013). Hippocampus, hippocampal sclerosis and epilepsy. Pharmacol Rep.

[11] Buckmaster PS (2004). Laboratory animal models of temporal lobe epilepsy. Comp Med.

[12] Levesque M, Avoli M, Bernard C (2016). Animal models of temporal lobe epilepsy following systemic chemoconvulsant administration. J Neurosci Methods.

[13] Gass P, Herdegen T, Bravo R, Kiessling M (1993). Spatiotemporal induction of immediate early genes in the rat brain after limbic seizures: effects of NMDA receptor antagonist MK-801. Eur J Neurosci.

[14] Herdegen T, Sandkuhler J, Gass P, Kiessling M, Bravo R, Zimmermann M (1993). JUN, FOS, KROX, and CREB transcription factor proteins in the rat cortex: basal expression and induction by spreading depression and epileptic seizures. J Comp Neurol.

[15] Hughes P, Dragunow M (1995). Induction of immediate-early genes and the control of neurotransmitter-regulated gene expression within the nervous system. Pharmacol Rev.

[16] Hughes PE, Alexi T, Walton M, Williams CE, Dragunow M, Clark RG, Gluckman PD (1999). Activity and injury-dependent expression of inducible transcription factors, growth factors and apoptosis-related genes within the central nervous system. Prog Neurobiol.

[17] Kiessling M, Gass P (1993). Immediate early gene expression in experimental epilepsy. Brain Pathol.

[18] Morimoto K, Fahnestock M, Racine RJ (2004). Kindling and status epilepticus models of epilepsy: rewiring the brain. Prog Neurobiol.

[19] Benito E, Barco A (2015). The neuronal activity-driven transcriptome. Mol Neurobiol.

[20] Flavell SW, Greenberg ME (2008). Signaling mechanisms linking neuronal activity to gene expression and plasticity of the nervous system. Annu Rev Neurosci.

[21] Knoll B, Nordheim A (2009). Functional versatility of transcription factors in the nervous system: the SRF paradigm. Trends Neurosci.

[22] Kuzniewska B, Nader K, Dabrowski M, Kaczmarek L, Kalita K (2016). Adult deletion of SRF increases Epileptogenesis and decreases activity-induced Gene expression. Mol Neurobiol.

[23] Etkin A, Alarcon JM, Weisberg SP, Touzani K, Huang YY, Nordheim A, Kandel ER (2006). A role in learning for SRF: deletion in the adult forebrain disrupts LTD and the formation of an immediate memory of a novel context. Neuron.

[24] Kalita K, Kharebava G, Zheng JJ, Hetman M (2006). Role of megakaryoblastic acute leukemia-1 in ERK1/2-dependent stimulation of serum response factor-driven transcription by BDNF or increased synaptic activity. J Neurosci.

[25] Meier C, Anastasiadou S, Knoll B (2011). Ephrin-A5 suppresses neurotrophin evoked neuronal motility, ERK activation and gene expression. Plos One.

[26] Ramanan N, Shen Y, Sarsfield S, Lemberger T, Schutz G, Linden DJ, Ginty DD (2005). SRF mediates activity-induced gene expression and synaptic plasticity but not neuronal viability. Nat Neurosci.

[27] Xia Z, Dudek H, Miranti CK, Greenberg ME (1996). Calcium influx via the NMDA receptor induces immediate early gene transcription by a MAP kinase/ERK-dependent mechanism. J Neurosci.

[28] Stern S, Knoll B (2014). CNS axon regeneration inhibitors stimulate an immediate early gene response via MAP kinase-SRF signaling. Mol Brain.

[29] Morris TA, Jafari N, Rice AC, Vasconcelos O, DeLorenzo RJ (1999). Persistent increased DNA-binding and expression of serum response factor occur with epilepsy-associated long-term plasticity changes. J Neurosci.

[30] Herdegen T, Blume A, Buschmann T, Georgakopoulos E, Winter C, Schmid W, Hsieh TF, Zimmermann M, Gass P (1997). Expression of activating transcription factor-2, serum response factor and cAMP/ca response element binding protein in the adult rat brain following generalized seizures, nerve fibre lesion and ultraviolet irradiation. Neuroscience.

[31] Cesari F, Brecht S, Vintersten K, Vuong LG, Hofmann M, Klingel K, Schnorr JJ, Arsenian S, Schild H, Herdegen T (2004). Mice deficient for the ets transcription factor elk-1 show normal immune responses and mildly impaired neuronal gene activation. Mol Cell Biol.

[32] Beck H, Flynn K, Lindenberg KS, Schwarz H, Bradke F, Di Giovanni S, Knoll B (2012). Serum response factor (SRF)-cofilin-actin signaling axis modulates mitochondrial dynamics. Proc Natl Acad Sci U S A.

[33] Stern S, Sinske D, Knoll B (2012). Serum response factor modulates neuron survival during peripheral axon injury. J Neuroinflammation.

[34] Knoll B, Kretz O, Fiedler C, Alberti S, Schutz G, Frotscher M, Nordheim A (2006). Serum response factor controls neuronal circuit assembly in the hippocampus. Nat Neurosci.

[35] Li CL, Sathyamurthy A, Oldenborg A, Tank D, Ramanan N (2014). SRF phosphorylation by glycogen synthase kinase-3 promotes axon growth in hippocampal neurons. J Neurosci.

[36] Lu PP, Ramanan N (2011). Serum response factor is required for cortical axon growth but is dispensable for neurogenesis and neocortical lamination. J Neurosci.

[37] Scandaglia M, Benito E, Morenilla-Palao C, Fiorenza A, Del Blanco B, Coca Y, Herrera E, Barco A (2015). Fine-tuned SRF activity controls asymmetrical neuronal outgrowth: implications for cortical migration, neural tissue lamination and circuit assembly. Sci Rep.

[38] Stern S, Haverkamp S, Sinske D, Tedeschi A, Naumann U, Di Giovanni S, Kochanek S, Nordheim A, Knoll B (2013). The transcription factor serum response factor stimulates axon regeneration through Cytoplasmic localization and Cofilin interaction. J Neurosci.

[39] Wickramasinghe SR, Alvania RS, Ramanan N, Wood JN, Mandai K, Ginty DD (2008). Serum response factor mediates NGF-dependent target Innervation by embryonic DRG sensory neurons. Neuron.

[40] Anastasiadou S, Liebenehm S, Sinske D, Meyer zu Reckendorf C, Moepps B, Nordheim A, Knoll B (2015). Neuronal expression of the transcription factor serum response factor modulates myelination in a mouse multiple sclerosis model. Glia.

[41] Boschert U, Muda M, Camps M, Dickinson R, Arkinstall S (1997). Induction of the dual specificity phosphatase PAC1 in rat brain following seizure activity. Neuroreport.

[42] Gass P, Eckhardt A, Schroder H, Bravo R, Herdegen T (1996). Transient expression of the mitogen-activated protein kinase phosphatase MKP-1 (3CH134/ERP1) in the rat brain after limbic epilepsy. Brain Res Mol Brain Res.

[43] Houser CR, Huang CS, Peng Z (2008). Dynamic seizure-related changes in extracellular signal-regulated kinase activation in a mouse model of temporal lobe epilepsy. Neuroscience.

[44] Li Y, Peng Z, Xiao B, Houser CR (2010). Activation of ERK by spontaneous seizures in neural progenitors of the dentate gyrus in a mouse model of epilepsy. Exp Neurol.

[45] Glazova MV, Nikitina LS, Hudik KA, Kirillova OD, Dorofeeva NA, Korotkov AA, Chernigovskaya EV (2015). Inhibition of ERK1/2 signaling prevents epileptiform behavior in rats prone to audiogenic seizures. J Neurochem.

[46] Jiang W, Van Cleemput J, Sheerin AH, Ji SP, Zhang Y, Saucier DM, Corcoran ME, Zhang X (2005). Involvement of extracellular regulated kinase and p38 kinase in hippocampal seizure tolerance. J Neurosci Res.

[47] Nateri AS, Raivich G, Gebhardt C, Da Costa C, Naumann H, Vreugdenhil M, Makwana M, Brandner S, Adams RH, Jefferys JG (2007). ERK activation causes epilepsy by stimulating NMDA receptor activity. EMBO J.

[48] Wiebel FF, Rennekampff V, Vintersten K, Nordheim A (2002). Generation of mice carrying conditional knockout alleles for the transcription factor SRF. Genesis.

[49] Erdmann G, Schutz G, Berger S (2007). Inducible gene inactivation in neurons of the adult mouse forebrain. BMC Neurosci.

[50] Chen J, Larionov S, Pitsch J, Hoerold N, Ullmann C, Elger CE, Schramm J, Becker AJ (2005). Expression analysis of metabotropic glutamate receptors I and III in mouse strains with different susceptibility to experimental temporal lobe epilepsy. Neurosci Lett.

[51] Pitsch J, Schoch S, Gueler N, Flor PJ, van der Putten H, Becker AJ (2007). Functional role of mGluR1 and mGluR4 in pilocarpine-induced temporal lobe epilepsy. Neurobiol Dis.

[52] Marques TE, de Mendonca LR, Pereira MG, de Andrade TG, Garcia-Cairasco N, Paco-Larson ML, Gitai DL (2013). Validation of suitable reference genes for expression studies in different pilocarpine-induced models of mesial temporal lobe epilepsy. Plos One.

[53] Irizarry RA, Hobbs B, Collin F, Beazer-Barclay YD, Antonellis KJ, Scherf U, Speed TP (2003). Exploration, normalization, and summaries of high density oligonucleotide array probe level data. Biostatistics.

[54] Meyer zu Reckendorf C, Anastasiadou S, Bachhuber F, Franz-Wachtel M, Macek B, Knoll B (2016). Proteomic analysis of SRF associated transcription complexes identified TFII-I as modulator of SRF function in neurons. Eur J Cell Biol.

[55] Schmued LC, Albertson C, Slikker W (1997). Fluoro-Jade: a novel fluorochrome for the sensitive and reliable histochemical localization of neuronal degeneration. Brain Res.

[56] Zambelli F, Pesole G, Pavesi G (2009). Pscan: finding over-represented transcription factor binding site motifs in sequences from co-regulated or co-expressed genes. Nucleic Acids Res.

[57] Szklarczyk D, Franceschini A, Wyder S, Forslund K, Heller D, Huerta-Cepas J, Simonovic M, Roth A, Santos A, Tsafou KP (2015). STRING v10: protein-protein interaction networks, integrated over the tree of life. Nucleic Acids Res.

[58] Zimprich A, Mroz G, Meyer Zu Reckendorf C, Anastasiadou S, Forstner P, Garrett L, Holter SM, Becker L, Rozman J, Prehn C, et al.: Serum Response Factor (SRF) Ablation Interferes with Acute Stress-Associated Immediate and Long-Term Coping Mechanisms. *Mol Neurobiol* 2016. doi:10.1007/s12035-016-0300-x 10.1007/s12035-016-0300-x 27914009

[59] Sun X, Lin Y (2016). Npas4: linking neuronal activity to memory. Trends Neurosci.

[60] Peng Z, Houser CR (2005). Temporal patterns of fos expression in the dentate gyrus after spontaneous seizures in a mouse model of temporal lobe epilepsy. J Neurosci.

[61] Vezzani A, Aronica E, Mazarati A, Pittman QJ (2013). Epilepsy and brain inflammation. Exp Neurol.

[62] Jessberger S, Parent JM: Epilepsy and Adult Neurogenesis. Cold Spring Harb Perspect Biol. 2015;7(12). 10.1101/cshperspect.a020677 PMC4665072 26552418

[63] Cho KO, Lybrand ZR, Ito N, Brulet R, Tafacory F, Zhang L, Good L, Ure K, Kernie SG, Birnbaum SG (2015). Aberrant hippocampal neurogenesis contributes to epilepsy and associated cognitive decline. Nat Commun.

[64] Rakhade SN, Shah AK, Agarwal R, Yao B, Asano E, Loeb JA (2007). Activity-dependent gene expression correlates with interictal spiking in human neocortical epilepsy. Epilepsia.

[65] Boschert U, Dickinson R, Muda M, Camps M, Arkinstall S (1998). Regulated expression of dual specificity protein phosphatases in rat brain. Neuroreport.

[66] Qian Z, Gilbert M, Kandel ER (1994). Temporal and spatial regulation of the expression of BAD2, a MAP kinase phosphatase, during seizure, kindling, and long-term potentiation. Learn Mem.

[67] Buckmaster PS, Zhang GF, Yamawaki R (2002). Axon sprouting in a model of temporal lobe epilepsy creates a predominantly excitatory feedback circuit. J Neurosci.

[68] Heng K, Haney MM, Buckmaster PS (2013). High-dose rapamycin blocks mossy fiber sprouting but not seizures in a mouse model of temporal lobe epilepsy. Epilepsia.

[69] Jin W, Zhang J, Lou D, Chavkin C, Xu M (2002). C-fos-deficient mouse hippocampal CA3 pyramidal neurons exhibit both enhanced basal and kainic acid-induced excitability. Neurosci Lett.

[70] Zhang J, Zhang D, McQuade JS, Behbehani M, Tsien JZ, Xu M (2002). C-fos regulates neuronal excitability and survival. Nat Genet.

[71] Hiroi N, Marek GJ, Brown JR, Ye H, Saudou F, Vaidya VA, Duman RS, Greenberg ME, Nestler EJ (1998). Essential role of the fosB gene in molecular, cellular, and behavioral actions of chronic electroconvulsive seizures. J Neurosci.

[72] Yutsudo N, Kamada T, Kajitani K, Nomaru H, Katogi A, Ohnishi YH, Ohnishi YN, Takase K, Sakumi K, Shigeto H, Nakabeppu Y (2013). fosB-null mice display impaired adult hippocampal neurogenesis and spontaneous epilepsy with depressive behavior. Neuropsychopharmacology.

[73] Mandel-Brehm C, Salogiannis J, Dhamne SC, Rotenberg A, Greenberg ME (2015). Seizure-like activity in a juvenile Angelman syndrome mouse model is attenuated by reducing arc expression. Proc Natl Acad Sci U S A.

[74] Lin Y, Bloodgood BL, Hauser JL, Lapan AD, Koon AC, Kim TK, Hu LS, Malik AN, Greenberg ME (2008). Activity-dependent regulation of inhibitory synapse development by Npas4. Nature.

[75] Zhang SJ, Zou M, Lu L, Lau D, Ditzel DA, Delucinge-Vivier C, Aso Y, Descombes P, Bading H (2009). Nuclear calcium signaling controls expression of a large gene pool: identification of a gene program for acquired neuroprotection induced by synaptic activity. Plos Genet.

[76] Zheng D, Butler LS, McNamara JO (1998). Kindling and associated mossy fibre sprouting are not affected in mice deficient of NGFI-A/NGFI-B genes. Neuroscience.

[77] Watanabe Y, Johnson RS, Butler LS, Binder DK, Spiegelman BM, Papaioannou VE, McNamara JO (1996). Null mutation of c-fos impairs structural and functional plasticities in the kindling model of epilepsy. J Neurosci.

[78] Buffet C, Catelli MG, Hecale-Perlemoine K, Bricaire L, Garcia C, Gallet-Dierick A, Rodriguez S, Cormier F, Groussin L (2015). Dual specificity Phosphatase 5, a specific negative regulator of ERK signaling, is induced by serum response factor and elk-1 transcription factor. Plos One.

[79] Patterson KI, Brummer T, O'Brien PM, Daly RJ (2009). Dual-specificity phosphatases: critical regulators with diverse cellular targets. Biochem J.

[80] Benito E, Valor LM, Jimenez-Minchan M, Huber W, Barco A (2011). cAMP response element-binding protein is a primary hub of activity-driven neuronal gene expression. J Neurosci.

[81] Vialou V, Maze I, Renthal W, LaPlant QC, Watts EL, Mouzon E, Ghose S, Tamminga CA, Nestler EJ (2010). Serum response factor promotes resilience to chronic social stress through the induction of DeltaFosB. J Neurosci.

