# Supplementary material for: SRF modulates seizure occurrence, activity induced gene transcription and hippocampal circuit reorganization in the mouse pilocarpine epilepsy model
Source: Mol Brain. 2017 Jul 17;10:30. doi: 10.1186/s13041-017-0310-2 (PMC5513048; doi:10.1186/s13041-017-0310-2)
Supplement: Supplementary file 2 — SRF ablation does not interfere with entering a kainic acid induced seizure period. (A, B) Wt and Srf mutant animals were injected with 20 μg/g bodyweight kainic acid (Tocris). Subsequently, seizures in mice were scored according to the modified Racine scale for approximately 2 h. Both, wt (black line) and SRF deficient (red line) animals developed grade 3–5 seizures and we did not observe an obvious difference between genotypes (A). In addition, the latency until a first grade 3 seizure was not affected by SRF ablation (B). Data are represented as mean ± SD. Number of animals are indicated or individual animals are labeled with grey circles. Figure S2. SRF ablation elevates numbers of DCX positive neurons. (A, B) Wt (A) and SRF deficient (B) sections of the dentate gyrus were labeled with anti. Doublecortin (DCX) directed antibodies. DCX positive neurons were localized to the dentate gyrus subgranular zone (A, B). In Srf mutant animals, numbers of DCX positive neurons were elevated compared to wt. (C) Quantification of DCX positive neurons per μm of the dentate gyrus. Data are represented as mean ± SD. Individual animals are labeled with grey circles or black squares. Scale-bar (A, B) = 100 μm. (PDF 713 kb) [file 13041_2017_310_MOESM2_ESM.pdf]

***SUPPLEMENTARY INFORMATIION***

# *SRF modulates seizure occurrence, activity induced gene transcription and hippocampal circuit reorganization in the mouse pilocarpine epilepsy model*

Pascal Lösing, Cristina Elena Niturad, Merle Harrer, Christopher Meyer zu Reckendorf, Theresa Schatz, Daniela Sinske, Holger Lerche, Snezana Maljevic and Bernd Knöll*

**Supplementary Materials & Methods**

**Kainic acid induced seizures**

Kainic acid was injected as described previously (Meyer zu Reckendorf et al., 2016). In short, wt and *Srf* mutant mice were injected intraperitoneally with 20 µg/g bodyweight kainic acid (Tocris) in NaCl, or the same volume of NaCl solution. Epileptic seizures were scored with the Racine scale (Racine, 1972) and sacrificed after 120 minutes: score 1 corresponds to immobility and facial clonus, score 2: head nodding; score 3: clonus of forelimbs; score 4: clonic seizures with falling and score 5: generalized tonic–clonic seizures.

**Immunohistochemistry**

Immediately after sacrificing the mice, the brains were washed with ice-cold PBS and fixed with 4% PFA/PBS at 4 °C for two days. Fixed brains were embedded in 4% agarose/PBS and 50 µm vibratome sections were prepared and washed 3 x 15 minutes with PBS at RT. After blocking for 30 minutes with PBST + 5% normal goat serum at RT, slides were incubated with an anti-DCX antibody (1:1000, Abcam) overnight at 4 °C. Brain slices were then washed 3 x 15 minutes with PBST and incubated for 30 minutes with Alexa 488 conjugated anti-rabbit secondary antibody (1:500, Molecular Probes), washed 3 x 15 minutes, counterstained with DAPI (AppliChem) and mounted with Mowiol (Calbiochem).

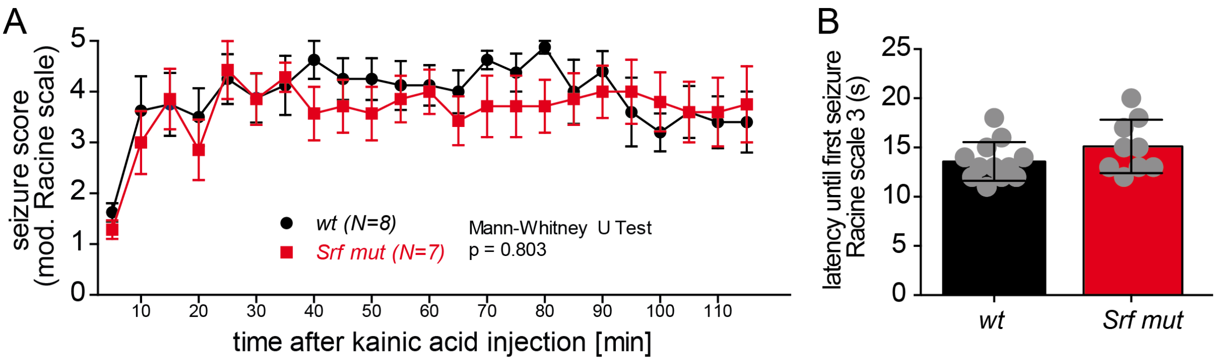


**Supplementary Figure 1**

**SRF ablation does not interfere with entering a kainic acid induced seizure period**

(A, B) Wt and *Srf* mutant animals were injected with 20 µg/g bodyweight kainic acid (Tocris). Subsequently, seizures in mice were scored according to the modified Racine scale for approximately two hours. Both, wt (black line) and SRF deficient (red line) animals developed grade 3-5 seizures and we did not observe an obvious difference between genotypes (A). In addition, the latency until a first grade 3 seizure was not affected by SRF ablation (B).

Data are represented as mean ± SD. Number of animals are indicated or individual animals are labeled with grey circles.

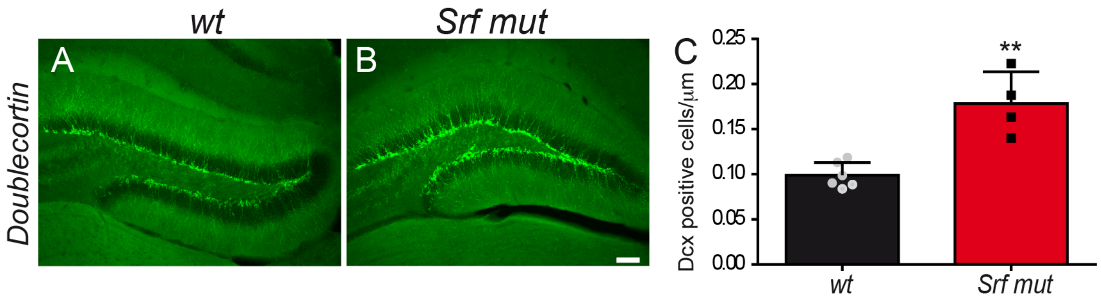


**Supplementary Figure 2**

**SRF ablation elevates numbers of DCX positive neurons**

(A, B) Wt (A) and SRF deficient (B) sections of the dentate gyrus were labeled with anti doublecortin (DCX) directed antibodies. DCX positive neurons were localized to the dentate gyrus subgranular zone (A, B). In Srf mutant animals, numbers of DCX positive neurons were elevated compared to wt.

(C) Quantification of DCX positive neurons per µm of the dentate gyrus. Data are represented as mean ± SD. Individual animals are labeled with grey circles or black squares.

Scale-bar (A, B) = 100 µm

**Supplemental References**

Meyer zu Reckendorf C, Anastasiadou S, Bachhuber F, Franz-Wachtel M, Macek B, Knoll B (2016) Proteomic analysis of SRF associated transcription complexes identified TFII-I as modulator of SRF function in neurons. European journal of cell biology 95:42-56.

Racine RJ (1972) Modification of seizure activity by electrical stimulation. I. After-discharge threshold. Electroencephalography and clinical neurophysiology 32:269-279.
